# Supplementary material for: A comprehensive but practical methodology for selecting biological indicators for long-term monitoring
Source: PLoS One. 2022 Mar 15;17(3):e0265246. doi: 10.1371/journal.pone.0265246 (PMC8923439; doi:10.1371/journal.pone.0265246)
Supplement: S1 Appendix — (DOCX) [file pone.0265246.s001.docx]

## S1 Appendix. DATA SOURCES

Alsina, A. (1986). Contribució al coneixement de l'entomofauna de la serra de l'Obac (O. Dermaptera, O. Dictioptera, O. Homoptera, O. Heteroptera, O. Coleoptera i O. Hymenoptera). PhD thesis, Universitat de Barcelona.

Andreu, J., Pino, J., Basnou, C., Guardiola, M. & Ordóñez, J.L. (2012) Les espècies exòtiques de Catalunya. Resum del projecte EXOCAT. Barcelona: CREAF.

Anglés, M., Badiella, E., Badiella, X., Casanovas, J. & Dalmases, C. (2001) Inventari de Cavitats de Sant Llorenç del Munt i l’Obac. Centre Excursionista de Terrassa, Secció d’Investigacions Subterrànies, Terrassa.Aparicio, E. (1997) Seguiment del barb cua-roig (*Barbus hasii*) a les rieres de Mura i Talamanca. Universitat de Barcelona, Barcelona.

Aparicio, E., Vargas, M.J. & Olmo, J.M. (1995) Estudi de les comunitats de peixos del Parc Natural de Sant Llorenç del Munt i l’Obac. Diputació de Barcelona, Barcelona.

Aparicio, E., Vargas, M.J. & Olmo, J.M., (2000). Avaluació de l’estat actual de la ictiofauna de les rieres del Parc Natural de Sant Llorenç del Munt i l’Obac. In: *IV Trobada d’Estudiosos de Sant Llorenç del Munt i l’Obac*: 97-101*.* Xarxa de Parcs Naturals (Ed.). Diputació de Barcelona: Barcelona.

Araujo, M.B., Pearson, R.G., Thuiller, W. & Erhard, M. (2005). Validation of species–climate impact models under climate change. **11**, 1504-1513.

Arnan, X. (2006). Dinàmica postincendi i interaccions entre plantes i formigues mediterrànies. PhD thesis, Universitat Autònoma de Barcelona.

Arnan, X., Cerdá, X., Rodrigo, A. & Retana, J. (2013). Response of ant functional composition to fire. **36**, 1182-1192.

Arnan, X., Rodrigo, A. & Retana, J. (2006). Post-fire recovery of Mediterranean ground ant communities follows vegetation and dryness gradients. *J. Biogeogr.,* **33**, 1246-1258.

Arnan, X., Rodrigo, A. & Retana, J. (2007). Post-fire regeneration of Mediterranean plant communities at a regional scale is dependent on vegetation type and dryness. **18**, 111-122.

Ayuso, M., Casadevall, S. & Mariné, R. (2008) Document estratègic de l’activitat cinegètica a la Xarxa de Parcs Naturals de la Diputació de Barcelona (Projecte: G020). 233*.* Diputació de Barcelona, Barcelona.

Badia, J., Bros, V., Germain, J., Gómez, A., Oms, O. & Vallès, F. (2008) Sant Llorenç del Munt: Les roques del Montcau i els roures de la canal de les Teixoneres. In: *Les sortides naturalistes de la institució.* Institució Catalana d'Història Natural, Barcelona.

Balaguer, L., Codina, J., Diego, F. & Sorolla, A., (1990). Estatus de la població de falcó pelegrí *Falco peregrinus* al Parc Natural de Sant Llorenç del Munt i l’Obac. In: *II Trobada d’Estudiosos de Sant Llorenç del Munt i l’Obac*: 37-38*.* Xarxa de Parcs Naturals (Ed.). Diputació de Barcelona: Barcelona.

Balcells, E. (1954). Quirópteros de cuevas catalanas: campaña de 1952-1953.

Balcells, E. (1964). Datos sobre la biología y migración del murciélago de cueva (*Miniopterus schreiberseii*, Chir. Vespert.) en el NE de España. **3**, 23-28.

Báldi, A. & Kisbenedek, T. (1997). Orthopteran assemblages as indicators of grassland naturalness in Hungary. **66**, 121-129.

Ballesteros, T. & Degollada, A. (1996) Estudi de l’avifauna al Parc Natural de Sant Llorenç del Munt. 44*.* Diputació de Barcelona, Barcelona.

Ballesteros, T. & Degollada, A., (2000). Estudi quantitatiu de l’avifauna del Parc Natural de Sant Llorenç del Munt i l’Obac. In: *IV Trobada d’Estudiosos de Sant Llorenç del Munt i l’Obac*: 107-111*.* Xarxa de Parcs Naturals (Ed.). Diputació de Barcelona: Barcelona.

Ballesteros, T. & Degollada, A., (2002). Dieta de la guineu (*Vulpes vulpes*) al Parc Natural de Sant Llorenç del Munt i l’Obac. In: *V Trobada d’Estudiosos de Sant Llorenç Munt i l’Obac*: 141-146*.* Xarxa de Parcs Naturals (Ed.). Diputació de Barcelona: Barelona, España.

Ballesteros, T., Degollada, A. & Baquedano, L. (1998). Estimación de la abundancia de zorro (*Vulpes vulpes*), garduñas (*Martes foina*) y gatos domésticos (*Felis catus*) en el P.N. de Sant Llorenç del Munt (Cataluña). **10**, 129-134.

Ballesteros, T., Degollada, A. & Baquedano, L., (2000a). Estudi dels carnívors al Parc Natural de Sant Llorenç del Munt i l’Obac. In: *IV Trobada d’Estudiosos de Sant Llorenç del Munt i l’Obac*: 113-118*.* Xarxa de Parcs Naturals (Ed.). Diputació de Barcelona: Barcelona.

Ballesteros, T., Degollada, A. & Plaza, V., (2000b). Dieta de la fagina al Parc Natural de Sant Llorenç del Munt i l’Obac. In: *IV Trobada d’Estudiosos de Sant Llorenç Munt i l’Obac*: 119-122*.* Xarxa de Parcs Naturals (Ed.). Diputació de Barcelona: Barcelona.

Ballesteros, T., Degollada, A. & Plaza, V., (2000c). Dieta de la geneta (*Genetta genetta*) al Parc Natural de Sant Llorenç del Munt i l’Obac. In: *IV Trobada d’Estudiosos de Sant Llorenç del Munt i l’Obac*: 123-125*.* Xarxa de Parcs Naturals (Ed.). Diputació de Barcelona: Barcelona.

Baró, F. & Lobo, A. (2007) Proposta de planificació del mosaic agroforestal del Parc Natural de Sant Llorenç del Munt i l’Obac. Una priorització dels Espais Oberts Agraris a partir d’una integració SIGAMC (Sistemes d’Informació Geogràfica – Avaluació Multicriteri). 127*.* Universitat Autònoma de Barcelona, Barcelona.

Barrientos, J.A., Nel·lo, M., Brañas, N., Mederos, J. & Masó, G. (2014a). Arañas rupícolas (*Arachnida, Araneae*) del Montcau (Barcelona, España). **24**, 65–73.

Barrientos, J.A., Nel·lo, M., Brañas, N., Mederos, J. & Masó, G., (2014b). L’aracnocenosi de les codines (el Montcau). In: *VIII Trobada d’Estudiosos de Sant Llorenç del Munt i l’Obac*: 83*.* Barcelona, D.d. (Ed.). Diputació de Barcelona: Barcelona.

Bas, J.M. & Gómez, C. (2003). Formigues dispersadores de llavors de *Rhamnus alaternus* (L.). **12**, 75-84.

Bas, J.M., Pons, P. & Gómez, C. (2006). Exclusive frugivory and seed dispersal of Rhamnus alaternus in the bird breeding season. *Plant Ecol.,* **183**, 77-89.

Bascompte, J., Jordano, P., Melián, C.J. & Olesen, J.M. (2003). The nested assembly of plant–animal mutualistic networks. **100**, 9383-9387.

Bécares, J. & Villero, D. (2002) Seguiment de les Poblacions de carvívors del Parc Natural de Sant Llorenç del Munt i l'Obac. 69*.* Diputació de Barcelona, Barcelona.

Beltrán, M., Piqué, M., Vericat, P. & Cervera, T. (2011) Models de gestió per als boscos de pi blanc (Pinus halepensis L.): producció de fusta i prevenció d’incendis forestals. Barcelona: Centre de la Propietat Forestal.

Beltrán, M., Vericat, P., Piqué, M. & Cervera, T. (2012) Models de gestió per als boscos de pinassa (Pinus nigra Arn.): producció de fusta i prevenció d’incendis forestals. Barcelona: Centre de la Propietat Forestal.

Bertoncelj, I. & Dolman, P.M. (2013). Conservation potential for heathland carabid beetle fauna of linear trackways within a plantation forest. **6**, 300-308.

Bogdziewicz, M. & Zwolak, R. (2014). Responses of small mammals to clear-cutting in temperate and boreal forests of Europe: a meta-analysis and review. *Eur. J. Forest Res.,* **133**, 1-11.

Boix, R., (1997). Els proturs d’un bosc mediterrani de la serra de l’Obac. In: *III Trobada d’estudiosos de Sant Llorenç del Munt i l’Obac. Monografies, 25*: 43-46*.* Xarxa de Parcs Naturals (Ed.). Diputació de Barcelona: Barcelona.

Bolòs, A. & Bolòs, O. (1950) Vegetación de las comarcas barcelonesas. Descripción geobotánica y catálogo florístico, según estudios efectuados por el propio autor y por Oriol de Bolos y Capdevila. Barcelona: Instituto Espafiol de Estudios Mediterráneos.

Bolòs, O. (1951). El elemento fitogeogràfico eurosiberiano en las sierras litorales catalanes. **3**, 1-42.

Bolòs, O. & Vigo, J. (1984) *Flora dela Països Catalans. Volum I*. Barcelona: Barcino.

Bolòs, O., Vigo, J., Masalles, R.M. & Ninot, J.M. (1990) *Flora manual dels països Catalans. Volum II*. Barcelona: Pòrtic SA.

Bosch, A. & Anglés, M. (2000) Itinerari general Sant Llorenç del Munt i Serra de l'Obac. 10*.* Historia natural.

Bouget, C., Larrieu, L. & Brin, A. (2014). Key features for saproxylic beetle diversity derived from rapid habitat assessment in temperate forests. **36**, 656-664.

Bouget, C., Lassauce, A. & Jonsell, M. (2012). Effects of fuelwood harvesting on biodiversity—a review focused on the situation in Europe. **42**, 1421-1432.

Brañas, N., Masó, G., Mederos-López, J. & Nel·lo, M. (2010) Estudi preliminar dels insectes i altres artròpodes a les codines del Parc Natural de Sant Llorenç del Munt i l’Obac. In: *Estudi de la biodiversitat de les codines del Montcau*: 45*.* Diputació de Barcelona, Àrea d'espais Naturals, Barcelona.

Brañas, N., Masó, G., Mederos-López, J. & Nel·lo, M. (2011) Continuació de l’estudi dels insectes i altres artròpodes a les codines del Parc Natural de Sant Llorenç del Munt i l’Obac. In: *Estudi de la biodiversitat de les codines del Montcau*: 45*.* Diputació de Barcelona, Àrea d'espais Naturals, Barcelona.

Bros, V., (2000). Els mol·luscs gasteròpodes (*Mollusca, Gasteropoda*) del massís de Sant Llorenç del Munt i la serra de l’Obac. In: *IV Trobada d’Estudiosos de Sant Llorenç del Munt i l’Obac*: 87-95*.* Xarxa de Parcs Naturals (Ed.). Diputació de Barcelona: Barcelona.

Bros, V., (2002). Presència de la nàiade *Anodonta cygnea* (Linné, 1758) (*Mollusca: Bivalvia, Unionidae*) al riu Ripoll. In: *V Trobada d’Estudiosos de Sant Llorenç del Munt i l’Obac*: 85-87*.* Xarxa de Parcs Naturals (Ed.). Diputació de Barcelona: Barcelona.

Bros, V. (2003) La selecció de l’hàbitat pels llimacs (*Gasteropoda: Pulmonata*) en el Parc Natural de Sant Llorenç del Munt i l’Obac. . In: *2a Sessió Científica sobre Invertebrats i Medi Ambient*: 6-7*.* Cervelló.

Bros, V. (2005) La recerca i la conservació de la fauna d’invertebrats en el Parc Natural de Sant Llorenç del Munt i l’Obac: tàxons d’ interès conservacionista 34*.* Diputació de Barcelona, Àrea d'espais Naturals, Barcelona.

Bros, V., (2008). Subprojecte Mol·luscs terrestres. In: Seguiment integral de la recolonització faunística post-incendi, a la zona afectada per l’incendi del 2003, al Parc Natural de Sant Llorenç del Munt i l’Obac: 119-134. Santos, X. (Ed.). Diputació de Barcelona: Barcelona.

Bros, V. (2010) Composició de la comunitat de mol·luscs de les codines en el Parc Natural de Sant Llorenç del Munt i l’Obac i l’impacte del trepig i l’erosió en el Montcau. In: *Estudi de la biodiversitat de les codines del Montcau*: 27*.* Diputació de Barcelona, Àrea d'espais Naturals, Barcelona.

Bros, V. & Guinart, D., (2002). Actualització del catàleg de la fauna vertebrada del Parc Natural de Sant Llorenç del Munt i l’Obac: recull bibliogràfic p reliminar. In: *V Trobada d’Estudiosos de Sant Llorenç del Munt i l’Obac. Monografies, 35*: 151-156*.* Xarxa de Parcs Naturals (Ed.). Diputació de Barcelona: Barcelons, España.

Bros, V. & Guinart, D., (2007). La recerca i la conservació de la fauna d’invertebrats al Parc Natural de Sant Llorenç del Munt i l’Obac. In: *VI Trobada d’Estudiosos de Sant Llorenç del Munt i l’Obac. Monografies,* : 99-107*.* Hernández, J., Grau, J. & Melero, J. (Eds.). Diputació de Barcelona: Barcelona.

Bros, V. & Llobet, T. (2011) Flora i fauna del Parc Natural Sant Llorenç del Munt i l'Obac (Guies il·lustrades de natura). Barcelona: Brau Edicions SL.

Bros, V., Miralles, J. & Real, J. (1980) Estudi de la fauna vertebrada del massís de Sant Llorenç del Munt i la Serra de l’Obac. Estudi encarregat com a base científica per a revisar el Pla Especial del Parc Natural de Sant Llorenç del Munt i l’Obac. Diputació de Barcelona, Barcelona.

Bros, V., Moreno-Rueda, G. & Santos, X. (2011). Does postfire management affect the recovery of Mediterranean communities? The case study of terrestrial gastropods. **261**, 611–619.

Bros, V., Torre, I. & Santos, X. (2016). Uncovering the environmental factors that influence diversity patterns of Mediterranean terrestrial Gastropod communities: a useful tool for conservation. *Ecol Res,* **31**, 39-47.

Brossa, L. (1999) Pla de seguiment de rapalòcers al Parc Natural de Sant Llorenç del Munt i serra de l’Obac. (Butterfly monitoring escheme). Diputació de Barcelona, Barcelona.

Buenestado, F.J., Ferreras, P., Blanco‐Aguiar, J.A., Tortosa, F.S. & Villafuerte, R. (2009). Survival and causes of mortality among wild Red‐legged Partridges Alectoris rufa in southern Spain: implications for conservation. *Ibis,* **151**, 720-730.

Buse, J., Assmann, T., Friedman, A.L.L., Rittner, O. & Pavlicek, T. (2013). Wood‐inhabiting beetles (Coleoptera) associated with oaks in a global biodiversity hotspot: a case study and checklist for Israel. **6**, 687-703.

Buse, J., Levanony, T., Timm, A., Dayan, T. & Assmann, T. (2010). Saproxylic beetle assemblages in the Mediterranean region: Impact of forest management on richness and structure. **259**, 1376-1384.

Bustamante Díaz, J. (1985). Food habits of the buzzard (*Buteo buteo* L. 1758) in the North of Spain. **12**, 51-62.

Butchart, S.H.M., Walpole, M., Collen, B., Van Strien, A., Scharlemann, J.P.W., Almond, R.E.A., Baillie, J.E.M., Bomhard, B., Brown, C. & Bruno, J. (2010). Global biodiversity: indicators of recent declines. **328**, 1164-1168.

Caballero, B., Masó, G., Mederos, J., Prieto, M., Brañas, N., Gago, S., Fadrique, F. & Auroux, L. (2019) Artròpodes del medi hipogeu del Parc Natural de Sant Llorenç del Munt i l’Obac. 66*.* Diputació de Barcelona, Barcelona.

Calderón, C.R. (1975). Sobre el papel de la perdiz roja (*Alectoris rufa*) en la dieta de los predadores ibéricos. **4**, 61-126.

Calvete, C. & Estrada, R. (2004). Short-term survival and dispersal of translocated European wild rabbits. Improving the release protocol. **120**, 507-516.

Calvete, C., Pelayo, E. & Sampietro, J. (2006). Habitat factors related to wild rabbit population trends after the initial impact of rabbit haemorrhagic disease. **33**, 467-474.

Campanaro, A., Bardiani, M., Spada, L., Carnevali, L., Montalto, F., Antonini, G., Mason, F. & Audisio, P. (2011) Linee guida per il monitoraggio e la conservazione dell’entomofauna saproxilica. Quaderni Conservazione Habitat, 6. In: *Quaderni Conservazione Habitat*: 44*.* MiPAAF, CFS, Verona, Italia.

Campeny, R. & Fernández, M. (2013) Tractament de les dades de seguiment de les poblacions d’amfibis del Parc Natural de Sant Llorenç del Munt i l’Obac. 48*.* MINUARTIA, Sant Celoni, España.

Camprodon, J. (2010). Biodiversitat i gestió forestal: estat de la qüestió. **44**, 10-17.

Camprodon, J. (2013) Ecologia i conservació dels ocells forestals: un manual de gestió de la biodiversitat en boscos catalans. Solsona: Centre Tecnològic Forestal de Catalunya.

Camprodon, J. & Brotons, L. (2006). Effects of undergrowth clearing on the bird communities of the Northwestern Mediterranean coppice holm oak forests. **221**, 72-82.

Camprodon, J. & Plana, E. (2001) Conservación de la biodiversidad y gestión forestal: su aplicación en la fauna vertebrada. Barcelona: Universitat de Barcelona.

Camprodon, J. & Plana, E. (2007) *Conservación de la biodiversidad, fauna vertebrada y gestión forestal*. Barcelona, España: Universitat de Barcelona.

Cañedo‐Argüelles, M., Fortuño, P., Prat, N., Vinyoles, D. & Bonada, N. (2019) Seguiment de la ictiofauna al Parc Natural de Sant Llorenç del Munt i l'Obac. 19*.* Diputació de Barcelona, Barcelona.

Cardona, F. (1990) Grans Cavitats de Catalunya. *Segon volum*: El sistema Mediterrani i la Depressió Central. Sant Llorenç del Munt i Serra de l’Obac. Espeleo Club de Gràcia.

Caritat, A. (1999) Producció de glans i virosta en diferents boscos del parc natural de la Zona Volcànica de La Garrotxa. 66*.* Olot.

Carles-Torlá, M. (2015). Algunos dípteros capturados en el Parque Natural de Sant Llorenç del Munt i l'Obac (Barcelona, España) (*Insecta*: *Diptera*). **15**, 75-77.

Carnicero, P. & Sáez, L. (2014) Inventariació de la flora al·lòctona de caràcter invasor del Parc de Sant Llorenç del Munt i Serra de l’Obac. 17*.* Diputació de Barcelona, Barcelona.

Carrera, D., Castell, C., Guinart, D., Loire, R., Torrentó, J., Vila-Escalé, M., Germain, J., Pino, J. & Real, J. (2015) Pla estratègic de seguiment i recerca per a la conservació del medi natural a la Xarxa de Parcs Naturals de la Diputació de Barcelona. 25*.* Diputació de Barcelona, Bercelona.

Carreras, J., Ferré, A. & Vigo, J. (2015) *Manual dels hàbitats de Catalunya*. Barcelona: Departament de Territori i Sostenibilitat.

Carrier, P. (2003). Effects of water addition on biotic and abiotic components of a dry boreal forest in the Yukon. PhD thesis, University of British Columbia.

Casas-Díaz, E., Peris, A., Serrano, E., Closa-Sebastià, F., Torrentó, J., Miño, À., Casanovas, R., Marco, I. & Lavín, S. (2012). Estima de la densidad de una población de jabalí (Sus scrofa) mediante trampeo fotográfico: estudio piloto en Cataluña. **23**, 99-104.

Castell, C., (2002). Efectos de la sequía en encinares del Parque Natural de Sant Llorenç del Munt i l’Obac (Barcelona). In: *V Trobada d’Estudiosos de Sant Llorenç del Munt i l’Obac*: 75-81*.* Xarxa de Parcs Naturals (Ed.). Diputació de Barcelona: Barcelona.

Castells, J. & Desclot, M. (1996) *Sant Llorenç del Munt i l’Obac*. Terrassa: Lunwerg Editores.

Catarina, A. (2007) Desarrollo de una aplicación para la gestión de la Fauna y Flora del Parque Natural de Sant Llorenç del Munt i l'Obac. Universitat Autonoma de Barcelona & Diputació de Barcelona (Ed.). Barcelona.

Cavia, V. (1990). Régimen alimenticio de la hormiga *Formica subrufa* (*Hymenoptera: Formicidae*). *Ses Entom ICHN-SCL,* **6**, 97-107.

CBMS (1994) Catalan Butterfly Monitoring Scheme (CBMS). Museu de Granollers Ciències Naturals, Granollers, Espanya.

Cerdà, X., Retana, J., Alsina, A. & Bosch, J., (1989). Estudi de les formigues (*Hymenoptera, Formicidae*) de la collada de les Tres Creus (serra de l’Obac). In: *I Trobada d’estudiosos de Sant Llorenç del Munt i l’Obac*: 71-75*.* Xarxa de Parcs Naturals (Ed.). Diputació de Barcelona: Barcelona.

Cid, N., Rieradevall, M., Ortíz, R., Cambra, J., Sostoa, A. & Prat, N., (2007). Qualitat ecològica de la riera de Mura en condicions de sequera. Anàlisi de les comunitats de macroinvertebrats, fitobentos i ictiofauna, del bosc de ribera i de l’hàbitat fluvial. In: *VI Trobada d’Estudiosos de Sant Llorenç del Munt i l’Obac*: 211-216*.* Hernández, J., Grau, J. & Melero, J. (Eds.). Diputació de Barcelona: Barcelona.

Cid, S., (2002). Macroinvertebrats aquàtics i qualitat ecològica de la riera de la Vall d’Horta. In: *V Trobada d’Estudiosos de Sant Llorenç del Munt i l’Obac. Monografies, 35*: 89-97*.* Xarxa de Parcs Naturals (Ed.). Diputació de Barcelona: Barcelona.

Cirera, M., (2002). Cens de la població d’astor (*Accipiter gentilis*) al Parc Natural de Sant Llorenç del Munt i l’Obac. In: *V Trobada d’Estudiosos de Sant Llorenç del Munt i l’Obac. Monografies, 35*: 35*.* Xarxa de Parcs Naturals (Ed.). Diputació de Barcelona: Barcelona.

Cirera, M. (2011) Seguiment de les poblacions de rapinyaires *Accipitriformes*, *Falconiformes* i *Estringiformes*, al Parc Natural de Sant Llorenç del Munt i l'Obac. 41*.* Barcelona.

Cirera, M., Gálvez, M. & Baqués, J.M., (2000). Estatus i distribució del falcó pelegrí (*Falco peregrinus*) al Parc Naturals de Sant Llorenç del Munt i l’Obac. In: *IV Trobada d’Estudiosos de Sant Llorenç del Munt i l’Obac*: 137*.* Xarxa de Parcs Naturals (Ed.). Diputació de Barcelona: Barcelona.

Cirera, M., Gálvez, M. & Ollé, À. (2002) Seguiment de les poblacions de rapinyaires Accipitriformes, Falconiformes i Estrigiformes, al Parc Natural de Sant Llorenç del Munt i l’Obac. 51*.* Barcelona.

Cirera, M. & Sorolla, A. (2000a) Aproximació a l’estat de les poblacions de rapinyaires diürns nidificants al Parc Natural de Sant Llorenç del Munt i serra de l’Obac. 43*.* Diputació de Barcelona, Barcelona.

Cirera, M. & Sorolla, A. (2000b) Cens de la població d’astor (*Accipiter gentilis*) al Parc Natural de Sant Llorenç del Munt i serra de l’Obac. 34*.* Diputació de Barcelona, Barcelona.

Clavero, M. & Brotons, L. (2010). Functional homogenization of bird communities along habitat gradients: accounting for niche multidimensionality. **19**, 684-696.

Clavero, M., Brotons, L. & Herrando, S. (2011). Bird community specialization, bird conservation and disturbance: the role of wildfires. **80**, 128–136.

Clavero, M., Brotons, L., Pons, P. & Sol, D. (2009). Prominent role of invasive species in avian biodiversity loss. *Biol Conserv,* **142**, 2043-2049.

Clavero, M., Hermoso, V., Levin, N. & Kark, S. (2010). Geographical linkages between threats and imperilment in freshwater fish in the Mediterranean Basin. **16**, 744–754.

Clavero, M., Prenda, J. & Delibes, M. (2005). Amphibian and reptile consumption by otters (Lutra lutra) in a coastal area in southern Iberian Peninsula. **15**, 125-131.

Closa-Sebastia, F., Casas-Diaz, E., Cuenca, R., Lavin, S., Mentaberre, G. & Marco, I. (2010). Brucella species antibodies and isolation in wild boar in north-east Spain. **167**, 826-828.

Closa-Sebastià, F., Casas-Díaz, E., Cuenca, R., Lavín, S., Mentaberre, G. & Marco, I. (2011). Antibodies to selected pathogens in wild boar (Sus scrofa) from Catalonia (NE Spain). **57**, 977-981.

Comellas, A., (2007). Apunts sobre roures i rouredes. In: *VI Trobada d’Estudiosos de Sant Llorenç del Munt i l’Obac*: 71-76*.* Hernández, J., Grau, J. & Melero, J. (Eds.). Diputació de Barcelona: Barcelona.

Comellas, A., (2009). Apunts sobre macrolíquens foliacis de Sant Llorenç del Munt. In: *VII Monografies de Sant Llorenç del Munt i l’Obac*: 152-155*.* Hernández, J., Grau, J. & Melero, J. (Eds.). Diputació de Barcelona: Barcelona.

Conill, G. & Mas, G. (2010) Conservació i seguiment del cranc de riu al Parc Natural de Sant Llorenç del Munt i l’Obac. Memòria 2009. 60*.* Diputació de Barcelona & Ecotons, Calldetenes.

Cruz, A., (1989). Isòpodes terrestres presents al massís de Sant Llorenç del Munt i serra de l’Obac (*Crustacea, Isopoda* i *Oniscidea*). In: *I Trobada d’estudiosos de Sant Llorenç del Munt i l’Obac*: 65-69*.* Xarxa de Parcs Naturals (Ed.). Diputació de Barcelona: Barcelona.

Díaz, M., Torre, I., Peris, A. & Tena, L. (2005). Foraging behavior of wood mice as related to presence and activity of genets. *J. Mammal.,* **86**, 1178-1185.

Diputació de Barcelona (2002) Aplicatiu de la base de dades de la fauna del Parc Natural de Sant Llorenç del Munt i l'Obac. Diputació de Barcelona (Ed.). Barcelona.

Diputació de Barcelona (2005) Sistema d'informació del parcel·lari del Parc Natural de Sant Llorenç del Munt i l'Obac. Diputació de Barcelona (Ed.). Barcelona.

Diputació de Barcelona (2014) Seguiment de flora amenaçada: Programa SEFA. Diputació de Barcelona, Àrea d'espais Naturals, Barcelona.

Dolsa, A. (1995) Estudi biogeogràfic dels lepidòpters heliòfils del Parc Natural de Sant Llorenç del Munt i serra de l’Obac. Diputació de Barcelona., Barcelona.

Donoso, I., Stefanescu, C., Martínez‐Abraín, A. & Traveset, A. (2016). Phenological asynchrony in plant–butterfly interactions associated with climate: a community‐wide perspective. **125**, 1434-1444.

Farrés, R., Vila, M., Prat, N., Gomà, J., Ortiz, R. & Cambra, J., (2007). Efectes d’un incendi forestal sobre la comunitat de fitobentos en una riera mediterrània. In: *VI Trobada d’Estudiosos de Sant Llorenç del Munt i l’Obac*: 263*.* Hernández, J., Grau, J. & Melero, J. (Eds.). Diputació de Barcelona: Barcelona.

FEM (2013) Efectes del Canvi Ambiental en les comunitats d’organismes dels RIus MEDiterranis (CARIMED). Freshwater Ecology amb Management (FEM) Research Group, Barcelona.

Fernández, C., Bladé, C. & Sauras, T., (2011). La regeneració natural de Pinus nigra subsp. salzmannii a la finca de can Dalmau del Parc Natural de Sant Llorenç del Munt i l'Obac. In: *VII Monografies de Sant Llorenç del Munt i l’Obac*: 177-191*.* Hernández, J., Grau, J. & Melero, J. (Eds.). Diputació de Barcelona: Barcelona.

Fernández-Ordóñez, J.C. (1999) Distribució dels ocells nidificants a la zona d’ampliació del Parc Natural de Sant Llorenç de Munt i serra de l’Obac. Diputació de Barcelona, Barcelona.

Fernández‐Chacón, A., Stefanescu, C., Genovart, M., Nichols, J.D., Hines, J.E., Paramo, F., Turco, M. & Oro, D. (2014). Determinants of extinction‐colonization dynamics in Mediterranean butterflies: the role of landscape, climate and local habitat features. **83**, 276-285.

Flaquer, C., Puig, X., Fàbregas, E., Guixé, D., Torre, I., Ràfols, R.G., Páramo, F., Camprodon, J., Cumplido, J.M., Ruíz-Jarillo, R., Baucells, A.L., Freixas, L. & Arrizabalaga, A. (2010). Revisión y aportación de datos sobre quirópteros de Catalunya: Propuesta de Lista Roja. **22**, 29-61.

Flaquer, C., Torre, I. & Arrizabalaga, A., (2007). Selección de refugios, gestión forestal y conservación de los quirópteros forestales. In: *Conservación de la biodiversidad y gestión forestal: su aplicación en la fauna vertebrada* 465-484*.* Camprodon, J. & Plana, E. (Eds.). Edicions de la Universitat de Barcelona: Barcelona.

Flaquer, V. & Segura, F. (1976). Contribució al coneixement del gènere *Stenasellus* Dolfus 1897 (*Crustacea, Isopoda*) de les cavitats subterrànies de Sant Llorenç del Munt - Serra de l’Obac. **6**, 31-36.

Font, X., de Cáceres, M., Quadrada, R.-V. & Navarro, A. (2000) Banc de Dades de Biodiversitat de Catalunya (BDBC). Generalitat de Catalunya & Universitat de Barcelona, Barcelona.

Fortuño, P., Acosta, R., Bonada, N., Cañedo-Argüelles, M., Castro, D., Cid, N., Múrria, C., Pineda, D., Rodríguez-Lozano, P., Soria, M., Tarrats, P., Verkaik, I. & Prat, N., (2019a). La disminució de les extraccions d’aigua millora l’estat hidrològic i ecològic del torrent de la Vall d’Horta. In: *IX Trobada d'Estudiosos de Sant Llorenç del Munt i l’Obac*: 250-261*.* Diputació de Barcelona (Ed.). Diputació de Barcelona: Barcelona.

Fortuño, P., Bonada, N., Prat, N., Acosta, R., Cañedo-Argüelles, M., Castro, D., Cid, N., Fernández, J., Gutiérrez-Cánovas, T., Múrria, C., Soria, M. & Verkaik, I. (2019b) Efectes del Canvi Ambiental en les comunitats d’organismes dels RIus MEDiterranis (CARIMED). Informe 2018-2019. In: *Estudis de la Qualitat Ecològica dels Rius*: 76*.* Diputació de Barcelona, Barcelona.

Francesc, O., Lago, P., Sabaté, S., Sauras, T. & Vallejo, R., (2007). Vulnerabilitat de la roureda de la Teixonera enfront del canvi climàtic. In: *Memòria anual de gestió del Parc Natural de Sant Llorenç del Munt i l’Obac*: 64*.* Xarxa de Parcs Naturals (Ed.). Diputació de Barcelon: Mura.

Gálvez, M., Cirera, M. & Baqués, J.M. (1998). Nidificación de Cárabo común *Sttrix aluco* en nido abandonado de Águila Perdicera *Hieraaetus fasciatus*. **15**, 43-45.

Gálvez, M., Cirera, M. & Baqués, J.M. (1999) Avaluació de les poblacions de rapinyaires nocturns al Parc Natural de Sant Llorenç del Munt i serra de l’Obac. 82*.* Diputació de Barcelona, Barcelona.

García, J.L.R. & Pujade-Villar, J. (2010). Descripción de una nueva especie de *Isolia* Förster (*Hymenoptera*: *Platygastridae*) del Nordeste Ibérico. **25**, 1-6.

Giménez, S., Guarner, N. & Giménez, M., (1993). Aportacions a l’estudi de *Salamandra salamandra* (L), *Amphibia Salamandridae*, a Sant Llorenç del Munt i l’Obac. Biometria. In: *III Trobada d’Estudiosos de Sant Llorenç del Munt i l’Obac. Monografies, 25*: 55*.* Xarxa de Parcs Naturals (Ed.). Diputació de Barcelona: Barcelona.

Goula, M., Navalpotro, H., Torres, L. & Ubach, A. (2014). On some interesting iberian true bugs (*Insecta*, *Hemiptera*, *Heteroptera*). **55**, 135–140.

Guardiola, M. & Gutiérrez, C. (2005) Base de dades de flora vascular del Parc Natural de Sant Llorenç del Munt i l’Obac. Actualització i millora 24*.* Diputació de Barcelona, Àrea d'espais Naturals, Barcelona.

Guardiola, M. & Gutiérrez, C. (2007) Pla de conservació de la flora vascular amenaçada al Parc natural de Sant Llorenç del Munt i l'Obac. Mesures d'estudi genèriques: cartografia de distribució dels tàxons al parc i fitxes tècniques dels tàxons. Diputació de Barcelona, Barcelona.

Gutiérrez, C. (2005) Pla de conservació de la flora vascular amenaçada al Parc Natural de Sant Llorenç del Munt i l’Obac. Llistat d’espècies. Definició dels criteris d’estudi i d’actuació. 55*.* Diputació de Barcelona, Barcelona.

Gutiérrez, C. & Guardiola, M. (2005) Base de dades de flora vascular del Parc Natural de Sant Llorenç del Munt i l’Obac. Diputació de Barcelona (Ed.). Barcelona.

Hernández, À.M., (2002). Aportació al coneixement dels pteridòfits del Parc Natural de Sant Llorenç del Munt i l’Obac. In: *V Trobada d’Estudiosos de Sant Llorenç del Munt i l’Obac. Monografies, 35*: 241-242*.* Xarxa de Parcs Naturals (Ed.). Diputació de Barcelona: Barcelona.

Hernández-Matías, A., Real, J., Moleón, M., Palma, L., Sánchez-Zapata, J.A., Pradel, R., Carrete, M., Gil-Sánchez, J.M., Beja, P. & Balbontín, J. (2013). From local monitoring to a broad‐scale viability assessment: A case study for the Bonelli's Eagle in western Europe. **83**, 239-261.

Hernández-Matías, A., Real, J., Pradel, R., Ravayrol, A., Vincent-Martin, N., Bosca, F. & Cheylan, G. (2010). Determinants of territorial recruitment in Bonelli's eagle (*Aquila fasciata*) populations. **127**, 173-184.

Herraiz, J.A. (2010). Estudio de las comunidades de hormigas de los diferentes tipos de vegetación del Parc Natural de Sant Llorenç del Munt i l'Obac. PhD thesis, Universidad Autónoma de Barcelona.

Herraiz, J.A. & Espadaler, X. (2009). Descripción de la reina de Lasius cinereus Seifert (Hymenoptera: Formicidae). **44**, 143-146.

Herraiz, J.A. & Espadaler, X., (2011). Estudi de les comunitats de formigues del mosaic de bosc de ribera del Parc Natural de Sant Llorenç del Munt i l’Obac. In: *VII Monografies de Sant Llorenç del Munt i l’Obac*: 53-61*.* Hernández, J., Grau, J. & Melero, J. (Eds.). Diputació de Barcelona: Barcelona.

Herrando, S., Anton, M., Sardà-Palomera, F., Bota, G., Gregory, R.D. & Brotons, L. (2014). Indicators of the impact of land use changes using large-scale bird surveys: Land abandonment in a Mediterranean region. **45**, 235-244.

Herrando, S. & Baltà, O., (2007). Patrons que determinen l’ocupació de caixes niu d’ocells a les àrees cremades a Sant Llorenç l’any 2003. In: *VI Trobada d’Estudiosos de Sant Llorenç del Munt i l’Obac*: 235-239*.* Hernández, J., Grau, J. & Melero, J. (Eds.). Diputació de Barcelona: Barcelona.

Herrando, S., Brotons, L., Anton, M., Páramo, F., Villero, D., Titeux, N., Quesada, J. & Stefanescu, C. (2016). Assessing impacts of land abandonment on Mediterranean biodiversity using indicators based on bird and butterfly monitoring data. **43**, 69-78.

Herrando, S., Brotons, L., Estrada, J., Guallar, S. & Anton, M. (2011) *Atles dels ocells de Catalunya a l'hivern 2006-2009*. Barcelona: Lynx Edicions & Institut Català d'Ornitologia.

Herrando, S., Brotons, L., Estrada, J. & Pedrocchi, V. (2008a). The Catalan Common Bird Survey (SOCC): a tool to estimate species population numbers. **24**, 138-146.

Herrando, S., Brotons, L., Guallar, S., Sales, S. & Pons, P. (2009). Postfire forest management and Mediterranean birds: the importance of the logging remnants. *Biodivers Conserv,* **18**, 2153-2164.

Herrando, S., Brotons, L. & Llacuna, S. (2003). Does fire increase the spatial heterogeneity of bird communities in Mediterranean landscapes? **145**, 307-317.

Herrando, S., Quesada, J. & Brotons, L., (2008b). Subprojecte: Aus. In: Seguiment integral de la recolonització faunística post-incendi, a la zona afectada per l’incendi del 2003, al Parc Natural de Sant Llorenç del Munt i l’Obac: 177-190. Santos, X. (Ed.). Diputació de Barcelona: Barcelona.

Herrando, S. & Sales, S., (2007). Efecte de la gestió forestal postincendi en l’avifauna de les àrees cremades a Sant Llorenç l’any 2003. In: *VI Trobada d’Estudiosos de Sant Llorenç del Munt i l’Obac*: 229-234*.* Hernández, J., Grau, J. & Melero, J. (Eds.). Diputació de Barcelona: Barcelona.

ICHN (2008) Invertebrats que requereixen mesures de conservació a Catalunya. 268*.* Institució Catalana d'Hisòria Natural, Barcelona.

ICHN (2010) Invertebrats que requereixen mesures de conservació a Catalunya. 14*.* Institució Catalana d'Hisòria Natural, Barcelona.

ICO (1991) Projecte SYLVIA. Institut Català d'Ornitologia (ICO), Barcelona.

ICO (2002) El Seguiment d'Ocells Comuns a Catalunya (SOCC). Institut Català d'Ornitologia (ICO), Barcelona.

ICO (2003) Ornitho.cat. Institut Català d'Ornitologia, Barcelona.

ICO (2013) Programa SOCC 2013. Programa de seguiment de l’avifauna al Parc Natural de Sant Llorenç del Munt i l’Obac. 40*.* Institut Català d’Ornitologia, Barcelona.

Joern, A. (1986). Experimental study of avian predation on coexisting grasshopper populations (Orthoptera: Acrididae) in a sandhills grassland. **46**, 243-249.

Jonas, J.L. & Joern, A. (2007). Grasshopper (Orthoptera: Acrididae) communities respond to fire, bison grazing and weather in North American tallgrass prairie: a long-term study. **153**, 699-711.

Julibert, L.J., (1990). Apunts de camp dels mamífers carnívors del Parc Natural de Sant Llorenç del Munt i l’Obac. In: *II Trobada d’Estudiosos de Sant Llorenç del Munt i l’Obac*: 49-51*.* Xarxa de Parcs Naturals (Ed.). Diputació de Barcelona: Barcelona.

Lassauce, A., Paillet, Y., Jactel, H. & Bouget, C. (2011). Deadwood as a surrogate for forest biodiversity: meta-analysis of correlations between deadwood volume and species richness of saproxylic organisms. **11**, 1027-1039.

Latorre, M., Real, M. & Alonso, M. (2007) Pla d’usos i gestió de la conca alta del riu Ripoll en l’àmbit del Parc Natural de Sant Llorenç del Munt i l’Obac. 52*.* Diputació de Barcelona, Barcelona.

Llop, E., Marí, T., Àlvaro, I. & Gómez-Bolea, A. (2010) Les criptògames com a indicadores del trepig a les codines del Montcau (Parc Natural de Sant Llorenç del Munt i l’Obac). In: *Estudi de la biodiversitat de les codines del Montcau*: 34*.* Diputació de Barcelona, Àrea d'espais Naturals, Barcelona.

Llop, E., Pinho, P., Matos, P., Pereira, M.J. & Branquinho, C. (2012). The use of lichen functional groups as indicators of air quality in a Mediterranean urban environment. **13**, 215-221.

Llorente, G.A., Clivillé, S., Montori, A., Santos, X. & Carretero, M.A. (1999) Determinació de punts d’aigua importants per a la reproducció dels amfibis a l’ampliació del Parc Natural de Sant Llorenç del Munt i Serra de l’Obac. Catàleg dels amfibis i dels rèptils. Diputació de Barcelona, Barcelona.

Llorente, G.A., Montori, A. & Albornà, P. (2002) Amfibis i rèptils del Parc Natural de Sant Llorenç del Munt i l’Obac. Universitat de Barcelona & Diputació de Barcelona (Ed.). Barcelona.

Llorente, G.A., Montori, A., Garriga, N., Richter-Boix, À. & Santos, X. (2006a) Incidència de les carreteres sobre els amfibis i rèptils als Parcs de Garraf, Sant Llorenç del Munt i l'Obac, Montnegre-Corredor i Montseny. Universitat de Barcelona (Ed.). Barcelona.

Llorente, G.A., Montori, A., Garriga, N., Richter-Boix, À. & Santos, X. (2006b) Incidència de les carreteres sobre els amfibis i rèptils als Parcs de Garraf, Sant Llorenç del Munt i Serra de l'Obac, Montnegte-Corredor i Montseny. 19*.* Departament de Biologia Animal, Universitat de Barcelona, Barcelona.

Llorente, G.A., Montori, A., Santos, X. & Carretero, M.A. (1995) *Atlas dels amfibis i rèptils de Catalunya i Andorra*. Figueres: El Brau.

Lobo, A., Ara, F., Martínez-Nieto, I. & Ferreruela, A., (2007). Impacte de l’incendi del Parc Natural de Sant Llorenç del Munt i l’Obac del 2003 i recuperació posterior: integrant informació remota i del terreny. In: *VI Trobada d’Estudiosos de Sant Llorenç del Munt i l’Obac.*: 44-49*.* Diputació de Barcelona: Barcelona.

López-Roig, M. & Serra-Cobo, J. (2014). Impact of human disturbance, density, and environmental conditions on the survival probabilities of pipistrelle bat (Pipistrellus pipistrellus). **56**, 471-480.

Maceda-Veiga, A., Basas, H., Lanzaco, G., Sala, M., Sostoa, A. & Serra, A. (2016). Impacts of the invader giant reed (*Arundo donax*) on riparian habitats and ground arthropod communities. **18**, 731-749.

Machordom, A., Berrebi, P. & Doadrio, I. (1990). Spanish barbel hybridization detected using enzymatic markers: *Barbus meridionalis* Risso × *Barbus haasi* Mertens (*Osteichthyes, Cyprinidae*). **3**, 295-303.

Mampel, T. (2012) Seguiment del status i la reproducció del Falcó pelegrí, Falco peregrinus, al Parc Natural de Sant Llorenç del Munt i l'Obac. Any 2011. 5*.* Diputació e Barcelona, Barcelona.

Mampel, T. & Bachs, L. (2013) Esperó de bolòs (*Delphinium bolosii*) al Parc Natural de Sant Llorenç del Munt. Distribució i cens de plantes reproductores, any 2013. 8*.* Diputació de Barcelona, Barcelona.

Mampel, T., Bros, V., Fàbrega, A., Torrentó, J. & Peris, A., (2014). Distribució i estatus dels rapinyaires forestals diürns al Parc Natural de Sant Llorenç del Munt i l'Obac. In: *VIII Trobada d'Estudiosos de Sant Llorenç del Munt i l'Obac*: 76-81*.* Duran, C., Hernández, J., Grau, J. & Melero, J. (Eds.). Diputació de Barcelona: Barcelona.

Marín, D., Olcina, V. & Nadal, J. (2001) Estudi de la qualitat dels hàbitats ecotònics mitjançant el seguiment d’espècies bioindicadores al Parc Natural de Sant Llorenç del Munt i l’Obac al llarg de l’any 2000. 31*.* Departament de Producció Animal, Universitat de Lleida, Lleida, España.

Martín-Nieto, I. (2003) Inventaris de sotabosc de les rouredes de Sant Llorenç del Munt. 65*.* Diputació de Barcelona, Barcelona.

Martínez, P. & Peris, A., (2009). Distribució i abundància del Gamarús (*Strix aluco*) al Parc Natural de Sant Llorenç del Munt i l’Obac. In: *VII Trobada d’Estudiosos de Sant Llorenç del Munt i l’Obac.* Hernández, J., Grau, J. & Melero, J. (Eds.). Diputació de Barcelona: Barcelona.

Martínez-Silvestre, A., Bertolero, A. & Soler-Massana, J. (2008) Programa de Conservació de les tortugues de rierol (*Mauremys leprosa*) i d’estany (*Emys orbicularis*), i de control de la tortuga de Florida (*Trachemys scripta* sp.) i d’altres quelonis al·lòctons a la Xarxa de Parcs Naturals de la Diputació de Barcelona. 126*.* CRARC scp, Diputació de Barcelona, Masquefa, España.

Massana-Canals, N., Arnal, J. & Pujade-Villar, J. (2013). Dades preliminars de la fauna secundària associada a gales de la forma asexual d'*Andricus hispanicus* (Hartig, 1856)(*Hymenoptera: Cynipidae*). *Butll Inst Cat Hist Nat,* **77**, 95-104.

Mateos, E. (1992). Colémbolos (Colembola, Insecta) edáficos de encinares de la Serra de l’Obac y la Serra de Prades (Sierra Prelitoral Catalana). Efectos de los incendios forestales sobre estos artrópodos. PhD thesis, Universitat de Barcelona.

Mateos, E., Parra, X., Sarlé, V. & Serra, A., (1994). Composició de la mesofauna d’artròpodes edàfics d’un alzinar de la serra de l’Obac. In: *II Trobada d’estudiosos de Sant Llorenç del Munt i l’Obac. Monografies, 21*: 53-58*.* Xarxa de Parcs Naturals (Ed.).

Mateos, E., Santos, X. & Pujade-Villar, J. (2011). Taxonomic and Functional Responses to Fire and Post-Fire Management of a Mediterranean Hymenoptera Community. **48**, 1000-1012.

Maynou, X. (2007-2009). Aportació al coneixement de la fauna odonatològica del massís de Sant Llorenç del Munt i la Serra de l'Obac. **75**, 85-98.

MCNG-DIBA (2015a) Pla estratègic de seguiment i recerca per la conservació de la fauna (ropalòcers, petits mamífers, quiròpters i amfibis) de la Xarxa de Parcs Naturals de la Diputació de Barcelona: PN-RB Montseny, PN Sant Llorenç del Munt i l’Obac, P.Garraf i Olèrdola, P. Montnegre i Corredor. Diputació de Barcelona (Ed.). Granollers, Espanya.

MCNG-DIBA (2015b) Pla estratègic de seguiment i recerca per la conservació de la fauna (ropalòcers, petits mamífers, quiròpters i amfibis) de la Xarxa de Parcs Naturals de la Diputació de Barcelona: PN-RB Montseny, PN Sant Llorenç del Munt i l’Obac, P.Garraf i Olèrdola, P. Montnegre i Corredor.: 129*.* Diputació de Barcelona, Granollers, Espanya.

Mederos-López, J., Nel·lo, M., Brañas, N., Caballero-López, B. & Masó, G., (2014). Estudi preliminar de la comunitat d’artròpodes de les codines del Parc Natural de Sant Llorenç del Munt i l’Obac. In: *VIII Trobada d’Estudiosos de Sant Llorenç del Munt i l’Obac*: 48-59*.* Duran, C., Hernández, J., Grau, J. & Melero, J. (Eds.). Diputació de Barcelona: Barcelona.

Mercadé, A. & Ferré, A. (2019) Cartografia digital dels hàbitats CORINE i dels Hàbitats d'Interès Comunitari del Parc Natural de Sant Llorenç del Munt i l'Obac a escala 1:10.000. 200*.* Universitat de Barcelona, Barcelona.

Miquel, C. & Serra, A., (1997). Primeres dades de l’estudi de la comunitat de macroartròpodes edàfics d’un alzinar de Sant Llorenç del Munt. In: *III Trobada d’estudiosos de Sant Llorenç del Munt i l’Obac. Monografies, 25*: 51-54*.* Xarxa de Parcs Naturals (Ed.). Diputació de Barcelona: Barcelona.

Miralles, E., Borràs, A., Campeny, R., Fernández, M., Fontanillas, M., Guardiola, M. & Planas, V. (2009) Pla especial de protecció del connector sur entre Sant Llorenç del Munt i Montserrat al municipi de Vacarisses. Ajuntament de Vacarisses & MINUARTIA, Vacarisses.

Miralles, J. (1982) Els ocells del parc natural de Sant Llorenç del Munt i serra de l’Obac. 23*.* Diputació de Barcelona, Barcelona.

Miralles, J. (1984). Presencia de *Fagus sylvatica* L. i d'altres especies tipiques de les rouredes humides i fagedes al massis de Sant Llorenç del Munt. **51**, 173-174.

Molero, J., Rovira, A., Simon, J., Bosch, M., López-Pujol, J., Massó, S. & Blanché, C. (2012) Avaluació del retrobament de Delphinium bolosii al Parc Natural de Sant Llorenç de Munt i de l’Obac (Fases 1 i 2). Propostes de gestió. METODOLOGIA. 29*.* Diputació de Barcelona, Barcelona.

Molero, J., Rovira, A., Simon, J., Bosch, M., López-Pujol, J., Massó, S. & Blanché, C. (2013) Avaluació del retrobament de Delphinium bolosii al Parc Natural de Sant Llorenç de Munt i de l’Obac (Fases 1 i 2). Propostes de gestió. INFORME FINAL. 52*.* Diputació de Barcelona, Barcelona.

Molero, J., Rovira, A., Simon, J., López-Pujol, J., Massó, S., Bosch, M. & Blanché, C. (2014a) Criteris i propostes de gestió a partir de l’estudi de biologia reproductiva de la població de Delphinium bolosii al Parc Natural de Sant Llorenç del Munt i l’Obac. EXTRACTE BÀSIC. 18*.* Diputació de Barcelona, Barcelona.

Molero, J., Rovira, A., Simon, J., López-Pujol, J., Massó, S., Bosch, M. & Blanché, C. (2014b) Criteris i propostes de gestió a partir de l’estudi de biologia reproductiva de la població de Delphinium bolosii al Parc Natural de Sant Llorenç del Munt i l’Obac. INFORME FINAL. 95*.* Diputació de Barcelona, Barcelona.

Montori, A., Clivillé, S., Llorente, G.A., Carretero, M.A. & Santos, X. (1999) La comunitat d’amfibis del Parc Natural de Sant Llorenç del Munt i l’Obac: catàleg i punts d’aigua importants per a la seva reproducció. 75*.* Diputació de Barcelona, Barcelona.

Montori, A., Clivillé, S., Llorente, G.A., Carretero, M.A. & Santos, X. (2002) La comunitat de rèptils del Parc Natural de Sant Llorenç del Munt i Serra de l’Obac. Catàleg i zones importants per a la seva conservació.: 45*.* Diputació de Barcelona, Barcelona.

Montori, A., Llorente, G.A., Carretero, M.A. & Santos, X., (2001). La gestión forestal en relación con la herpetofauna. In: *Conservación de la biodiversidad y gestión forestal: su aplicación en la fauna vertebrada*: 251-289*.* Camprodon, J. & Plana, E. (Eds.). Edicions de la Universitat de Barcelona: Barcelona.

Munné, A. & Prat, N. (2011). Effects of Mediterranean climate annual variability on stream biological quality assessment using macroinvertebrate communities. **11**, 651-662.

Munné, A., Solà, C. & Prat, N. (1998). QBR: Un índice rápido para la evaluación de la calidad de los ecosistemas de ribera. **175**, 20-37.

Muñoz, Q. (2012) Actualització de les dades recollides a l’estació del Catalan Butterfly Monitoring Scheme (CBMS) al Parc Natural de Sant Llorenç del Munt i L’Obac. Temporada 2012. 35*.* Museu de Granollers Ciències Naturals & Diputació de Barcelona, Granollers.

Muñoz, Q., Stefanescu, C. & Jubany, J. (2012) El Seguiment dels Ropalòcers al Parc Natural de Sant Llorenç del Munt i l’Obac. Temprada 2011. 44*.* Museu de Granollers Ciències Naturals & Diputació de Barcelona, Granollers.

Nebot, M., Hernàndez-Ruiz, T., Panareda, J.M., Pintó, J., Boccio, M., Orús, E. & Badia, A., (2014). Introducció a l’estudi de les crassulàcies de Sant Llorenç del Munt i l’Obac. In: *VIII Trobada d’Estudiosos de Sant Llorenç del Munt i l’Obac*: 178-183*.* Duran, C., Hernández, J., Grau, J. & Melero, J. (Eds.). Diputació de Barcelona: Barcelona.

Olmo-Vidal, J.M. (2006) *Atles del ortòpters de Catalunya i llibre vermell: Llagostes, saltamartins, grills, someretes,* 2nd edn. Barcelona: Generalitat de Catalunya. Departament de Mediambient i Habitatge.

Oxygastra (2007a) Informe de les activitats de camp realitzades pel grup de treball Oxygastra al massís de Sant Llorenç del Munt i l’Obac. Institució Catalana d’Història Natural (Ed.). Barcelona.

Oxygastra (2007b) Informe de les activitats de camp realitzades pel grup de treball Oxygastra al massís de Sant Llorenç del Munt i l’Obac. 7*.* Institució Catalana d’Història Natural, Barcelona.

Pace, G., Acosta, R., Rieradevall, M., Fortuño, P. & Prat, N. (2013) Nimfes d’EFEMERÒPTERS dels rius Llobregat i Besòs. Guia d’identificació dels gèneres i de les espècies més comunes. 18*.* Freshwater Ecology and Management Research Group, Barcelona.

Palou, A., Casas, C. & Sáez, L., (2014). Problemàtica de conservació de l’endemisme del massís de Sant Llorenç del Munt Arenaria fontqueri ssp. cavanillesiana (Caryophyllaceae). In: *VIII Trobada d’Estudiosos de Sant Llorenç del Munt i l’Obac*: 184-192*.* Duran, C., Hernández, J., Grau, J. & Melero, J. (Eds.). Diputació de Barcelona: Barceloan, España.

Palou, A. & Sáez, L. (2011) Estudi demogràfic, corològic i de l’estat de conservació de l’endemisme de Sant Llorenç del Munt i de la Serra de l’Obac *Arenaria fontqueri* subsp. *cavanillesiana*. 17*.* Diputació de Barcelona, Àrea d'espais Naturals.

Panareda, J.M. & Pintó, J. (1997) *Sant Llorenç del Munt: Visió geogràfica*. Vic, Barcelona: Eumo Editorial.

Paricio, S., (2007). Comparació d’un sòl cremat i d’un de no cremat de l’incendi de Sant Llorenç del Munt i l’Obac de l’agost del 2003. In: *VI Trobada d’Estudiosos de Sant Llorenç del Munt i l’Obac*: 253-259*.* Hernández, J., Grau, J. & Melero, J. (Eds.). Diputació de Barcelona: Barcelona.

Parsons, P.A. (1991). Biodiversity conservation under global climatic change: the insect Drosophila as a biological indicator? . **1**, 77–83.

Pasquina, À., Carretero, M.Á. & Colomer, T. (1999) Seguiment de les poblacions de carnívors de la zona d’ampliació del Parc Natural de Sant Llorenç del Munt i l’Obac. 63*.* Diputació de Barcelona, Barcelona.

Pastor, J., (2014). Recerca i estudi del registre de la fauna invertebrada descoberta a les cavitats del Parc de Sant Llorenç del Munt i l’Obac. In: *VIII Trobada d’Estudiosos de Sant Llorenç del Munt i l’Obac*: 42-47*.* Duran, C., Hernández, J., Grau, J. & Melero, J. (Eds.). Diputació de Barcelona: Barcelona.

Pausas, J.G. (2004). Changes in fire and climate in the eastern Iberian Peninsula (Mediterranean basin). **63**, 337-350.

Pausas, J.G., Keeley, J.E. & Schwilk, D.W. (2017). Flammability as an ecological and evolutionary driver. **105**, 289-297.

Perera, A., (1989). Estudi dels aràcnids (escorpins, pseudo-escorpins, opilion) d’un alzinar mediterrani muntanyenc: la serra de l’Obac. In: *I Trobada d’Estudiosos de Sant Llorenç del Munt i l’Obac*: 51-56*.* Xarxa de Parcs Naturals (Ed.). Diputació de Barcelona: Barcelona.

Peris, A. (2019). *Ecologia del senglar (Sus scrofa) en ambients mediterranis*. PhD thesis, Universitat Autànoma de Barcelona.

Peris, A., Bros, V. & Torrentó, J. (2007) Resultats del Cens de conill (*Oryctolagus cuniculus*) al Parc Natural de Sant Llorenç del Munt i l’Obac. 4*.* Diputació de Barcelona, Barcelona.

Peris, A., Bros, V. & Torrentó, J., (2011a). Bases metodològiques per al seguiment de les poblacions de conill (*Oryctolagus cuniculus*) al Parc Natural de Sant Llorenç del Munt i l’Obac. In: *VII Trobada Estudiosos de Sant Llorenç del Munt i l’Obac*: 81-85*.* Hernández, J., Grau, J. & Melero, J. (Eds.). Diputació de Barcelona: Barcelona.

Peris, A. & Campuzano, R., (2011). Els carnívors al Parc Natural de Sant Llorenç del Munt i l’Obac. Estudi mitjançant trampeig fotogràfic. In: *VII Monografies de Sant Llorenç del Munt i l’Obac*: 86-94*.* Hernández, J., Grau, J. & Melero, J. (Eds.). Diputació de Barcelona: Barcelona.

Peris, A., Casas-Díaz, E., Closa-Sebastià, F., Marco, I., Lavín, S. & Miño, À. (2013) Aproximació metodològica i avaluació de l’estat poblacional del Cabirol (*Capreolus capreolus*) al Parc Natural de Sant Llorenç del Munt i l’Obac. 21*.* Diputació de Barcelona, Barcelona.

Peris, A., Casas-Díaz, E., Closa-Sebastià, F., Marco, I., Lavín, S. & Miño, À., (2014a). Avaluació de l’estat poblacional del cabirol (*Capreolus capreolus*) al Parc Natural de Sant Llorenç del Munt i l’Obac. In: *VIII Trobada d’Estudiosos de Sant Llorenç del Munt i l’Obac*: 84*.* Duran, C., Hernández, J., Grau, J. & Melero, J. (Eds.). Diputació de Barcelona: Barcelona.

Peris, A., Closa-Sebastià, F., Marco, I., Lavín, S., Miño, À., Torrentó, J. & Casas-Díaz, E., (2014b). Identificació de senglars (*Sus scrofa*) al Parc Natural de Sant Llorenç del Munt i l’Obac i feed-back d’informació proporcionat durant cinc anys de seguiment. In: *VIII Trobada d’Estudiosos de Sant Llorenç del Munt i l’Obac*: 85*.* Duran, C., Hernández, J., Grau, J. & Melero, J. (Eds.). Diputació de Barcelona: Bareclona, España.

Peris, A. & Mampel, T. (2010) Seguiment de la població nidificant de mussol banyut (Asio otus) i gamarús (Strix aluco) al Parc de Sant Llorenç del Munt i L’Obac. 15*.* Xarxa de Parcs Naturals, Diputació de Barcelona, PN Sant Llorenç del Munt i l'Obac, España.

Peris, A. & Mampel, T. (2011) Proposta de Seguiment de dels Rapinyaires Nocturns al Parc Natural de Sant Llorenç del Munt i l’Obac. 15*.* Xarxa de Parcs Naturals, Diputació de Barcelons, PN Sant Llorenç del Munt i l'Obac, España.

Peris, A., Tena, L. & Campuzano, R. (2008) La comunitat de carnívors del Parc Natural de Sant Llorenç del Munt i l’Obac. Respostes a la gestió, distribució i selecció d’hàbitat. 42*.* Diputació de Barcelona, Àrea d'espais Naturals, Barcelona.

Peris, A., Tena, L. & Villena, A. (2011b). Abundancia de ginetas (*Genetta genetta*) en un encinar mediterráneo. Estimación mediante trampeo fotográfico. **23**, 73-79.

Pintó, J. (1990). Cartografia de la vegetació de St. Llorenç del Munt. **19**, 57-72.

Pintó, J. (1992). Els boscos de Sant Llorenç del Munt. **20-21**, 147-156.

Pintó, J. (1993). La Dinàmica de la vegetació a St. Llorenç del Munt. **22**, 25-54.

Pintó, J., (1997a). Flora i corologia de les plantes vasculars de Sant Llorenç del Munt i l'Obac: les estepes (gènere Cistus). In: *III Trobada d’estudiosos de Sant Llorenç del Munt i l’Obac. Monografies, 25*: 17-21*.* Barcelona, D.d. (Ed.). Diputació de Barcelona: Barcelona.

Pintó, J., (1997b). Vegetació de Sant Llorenç del Munt: les comunitats forestals de la part alta del massís. In: *Trobada d'estudiosos de Sant Llorenç del Munt i l'Obac*: 23-26*.* Xarxa de Parcs Naturals (Ed.). Diputació de Barcelona: Barcelona.

Pintó, J., (2000). Flora i corologia de les plantes vasculars de Sant Llorenç del Munt i l'Obac: els pins. In: *IV Trobada d’Estudiosos de Sant Llorenç del Munt i l’Obac*: 29-32*.* Barcelona, D.d. (Ed.). Diputació de Barcelona: Barcelona.

Pintó, J. & Panareda, J.M. (1995) Memòria del mapa de la vegetació de Sant Llorenç del Munt. Barcelona: Aster.

Piqué, M., Beltrán, M., Vericat, P., Calama, R. & Cervera, T. (2015) Models de gestió per a les pinedes de pi pinyer (Pinus pinea L.): producció de fusta i pinya i prevenció d’incendis forestals. Barcelona: Centre de la Propietat Forestal.

Piqué, M., Beltrán, M., Vericat, P., Cervera, T., Farrio, R. & Baiges, T. (2011) *Models de gestió per als boscos de pi roig (Pinus sylvestris L.): producció de fusta i prevenció d’incendis forestals*. Barcelona: Centre de la Propietat Forestal.

Pla, M., Solórzano, S. & Villero, D. (2001) Base de dades d'artròpodes en el Parc Natural de Sant Llorenç del Munt i l'Obac. Diputació de Barcelona (Ed.). Barcelona.

Pla, M., Solórzano, S. & Villero, D. (2002) Recopilació bibliogràfica de citacions d’Artròpodes en el Parc Natural de Sant Llorenç del Munt i l’Obac. Diputació de Barcelona, Barcelona.

Prat, N., Bonada, N. & Rieradevall, M. (2000) Estat ecològic del Torrent de Castelló. Diputació de Barcelona, Barcelona.

Prat, N., Fortuño, P. & Rieradevall, M. (2009a) Manual d’utilització de L’Índex d’Hàbitat Fluvial (IHF). 26*.* Diputació de Barcelona, Barcelona.

Prat, N., Fortuño, P., Rieradevall, M., Acosta, R., Bonada, N., Castro, D., Cañedo-Argüelles, M., Cid, N., Múrria, C., Rodríguez-Lozano, P., Sánchez, N. & Tarrats, P. (2015) Efectes del Canvi Ambiental en les comunitats d’organismes dels RIus MEDiterranis (CARIMED). Informe 2015. In: *Estudis de la Qualitat Ecològica dels Rius*: 86*.* Diputació de Barcelona, Barcelona.

Prat, N., Fortuño, P., Rodríguez, P. & Rieradevall, M., (2014). Efectes de l’extracció d’aigua sobre la comunitat de macroinvertebrats al Parc Natural de Sant Llorenç del Munt i l’Obac. In: *VIII Trobada d’Estudiosos de Sant Llorenç del Munt i l’Obac*: 166*.* Duran, C., Hernández, J., Grau, J. & Melero, J. (Eds.). Diputació de Barcelona: Barcelona.

Prat, N. & Rieradevall, M. (1996) La qualitat biològica de les aigües del Parc Natural de Sant Llorenç del Munt i de la Serra de l’Obac. Departament d’Ecologia, Universitat de Barcelona, Barcelona.

Prat, N., Rieradevall, M. & Fortuño, P. (2012) Metodologia F.E.M. per a l’avaluació de l’estat ecològic dels rius Mediterranis. 44*.* Universitat de Barcelona, Barcelona.

Prat, N., Rieradevall, M., Munné, A., Solá, C. & Chacon, G. (1997) La qualitat ecològica del Besòs i el Llobregat. In: *Estudis de la Qualitat Ecològica dels Rius*: 153*.* Diputació de Barcelona, Barcelona.

Prat, N., Rieradevall, M., Vila-Escalé, M., Vegas, T., Verkaik, I., Sostoa, A., Vinyoles, D., Caiola, N., Maceda, A., Peiró, L., Farrés, R., Gomà, J. & Cambra, J., (2007). Diagnosi dels efectes del foc forestal de l’estiu del 2003 en els ecosistemes aquàtics del riu Ripoll i proposta de mesures de restauració. In: *VI Trobada d’Estudiosos de Sant Llorenç del Munt i l’Obac*: 265*.* Hernández, J., Grau, J. & Melero, J. (Eds.). Diputació de Barcelona: Barcelona.

Prat, N., Ríos, B., Acosta, R. & Rieradevall, M., (2009b). Los macroinvertebrados como indicadores de calidad de las aguas. In: *Macroinvertebrados bentónicos sudamericanos. Sistemática y biología*: 631-654*.* Domínguez, E. & Fernández, H.R. (Eds.). Fundación Miguel Lillo: Tucumán, Argentina.

Prat, N., Vegas, T., Rieradevall, M. & Vila-Escalé, M. (2004) Efectes del foc forestal de Gallifa sobre l’estat ecològic de la seva riera. In: *Acció Especial (ACES 03).* 37*.* Universitat de Barcelona, Barcelona.

Puig-Gironès, R. (2016). Estudi dels patrons de colonització postincendi en vertebrats al llarg de gradients ambientals i espacials. PhD thesis, University of Girona.

Puig-Gironès, R., Brotons, L. & Pons, P. (2017). Aridity influences the recovery of Mediterranean shrubland birds after wildfire. *PLoS ONE,* **12**, e0173599.

Pujade-Villar, J. (1985). Sobre alguns cinípids cecidògens trobats en els roures de Sant Llorenç del Munt i serra de l’Obac. **1**, 87-92.

Pujade-Villar, J. (1993). Sobre els Megastigmus Dalman (Hymenoptera: Chalcidoidea, Torymidae) detectats a Catalunya a partir de la recol·lecció de Cecidis de cinípids produïts sobre Rosa i Quercus. **VII**, 53-58.

Pujade-Villar, J. (1994a). Complex parasitari de Myopites limbardae Schiner, 1864 (Diptera: Tephritidae) detectat al nord-est ibèric (Hym.: Eupeliviidae, Pteromalidae, Torymidae, Eurytoivhdae). **VIII**, 49-60.

Pujade-Villar, J. (1994b). Sobre las especies de pteromálidos (Hym., Pteromalidae) detectadas en agallas de Diplolepis sp. (Hym., Cynipidae) en Cataluña. **9**, 107-108.

Pujade-Villar, J. (1994c). Sobre les especies de Sycophila Walker, 1871 relacionades amb cinipo-cecidis recol·lectats sobre Quercus i Rosa a Catalunya (Hymenoptera: Chalcididae: Eurytomidae). 69-79.

Pujol-Buxó, E. (2019) Validació de la Metodologia per al Seguiment de Rèptils al Parc del Garraf i al Parc Natural de Sant Llorenç del Munt i l’Obac. 44*.* Diputació de Barcelona, Barcelona.

Rambla, M. (1977). Opilions (*Arachnida*) de les cavitats de Sant Llorenç del Munt- Serra de l'Obac. Comunicacions del 6è simposium d'espeleologia. Terrassa. **6**, 9-16.

Real, J. (1981). Aproximació a l’estudi dels rapinyaires (*Falcociformes*) dels massissos de Sant Llorenç del Munt-Serra de l’Obac, Montserrat i zones envoltants. **47**, 155-164.

Real, J. (1982). Contribució al coneixement de la biologia i distribució de l’àliga cuabarrada (Hieraaetus fasciatus, Vieillot, 1822) a la Serralada pre-litoral catalana (Falconiformes, Accipitridae). PhD thesis, Universitat autònoma de Barcelona.

Real, J. (1983). Addicions a l’estudi dels rapinyaires (*Falconiformes*) dels massisos de Sant Llorenç del Munt-Serra de l’Obac, Montserrat i zones envoltants. **49 (Sec. Zool. 5)**, 155-158.

Real, J. (1985a) Estudi de les espècies cinegètiques (*Oryctolagus cuniculus*, *Alectoris rufa* i *Sciurus vulgaris*) i control i protecció de l’àliga cuabarrada *Hieraaetus fasciatus*, al Parc Natural de Sant Llorenç del Munt i Serra de l’Obac. 39*.* Diputació de Barcelona, Barcelona.

Real, J., (2004). Aguila azor-perdicera, *Hieraaetus fasciatus*. In: *Libro Rojo de las Aves de España*: 154–157*.* Madroño, A., Gonzàlez, C. & Atienza, J. (Eds.). Dirección General para la Biodiversidad-SEO/Birdlife: Madrid, Spain.

Real, J., Bosch, R., Hernández-Matías, A. & Tintó, A. (2007) Modelització de la població d’àliga perdiguera *Hieraaetus fasciatus* a la província de Barcelona en el marc del programa SITXELL. 123*.* Diputació de Barcelona, Barcelona.

Real, J., Bosch, R., Hernández-Matías, A. & Tintó, A. (2008) Seguiment de la població d’àliga perdiguera (*Hieraaetus fasciatus*) a la província de Barcelona i anàlisi de la qualitat dels hàbitats en el marc del projecte SITXELL. 144*.* Diputació de Barcelona, Barcelona.

Real, J. & Bros, V. (1983) Memòria-proposta per a l’estudi i ajut de l’àliga cuabarrada (*Hieraaetus fasciatus*) al Parc Natural de Sant Llorenç del Munt i l’Obac. Diputació de Barcelona, Barcelona.

Real, J. & Bros, V., (1989). L’inventari dels vertebrats de Sant Llorenç del Munt i serra de l’Obac. Relacions biogeogràfiques amb el Montseny, Garraf, Montnegre, Collserola i Montserrat. In: *I Trobada d’Estudiosos de Sant Llorenç del Munt i l’Obac*: 33-41*.* Xarxa de Parcs Naturals (Ed.). Diputació de Barcelona: Barcelona.

Real, J., Galobart, À. & Fernández, J. (1985). Estudi preliminar d’una població de duc *Bubo bubo* al Vallès i Bages. **1**, 175-187.

Real, J., Hernández-Matías, A., Rollan, À. & Tintó, A. (2015) El Águila perdicera en Cataluña: de la amenaza a la conservación aplicaciones a la mitigación de la electrocución. Barceloana, España: ENDESA, S.A.

Real, J. & Ribas, J. (1985). Status, distribució i migració dels rapinyaires diürns (*Falconiformes* i *Accipitriformes*) al Vallès Occidental i Oriental. **1**, 151-170.

Real, M. (1985b). Aproximació als macroinvertebrats d’aigua dolça del massís de Sant Llorenç del Munt. **1**, 79-86.

Real, M., Rieradevall, M. & Prat, N., (1989). Limnologia de les rieres del massís de Sant Llorenç del Munt i l’Obac. In: *I Trobada d’estudiosos de Sant Llorenç del Munt i l’Obac*: 79-81*.* Xarxa de Parcs Naturals (Ed.). Diputació de Barcelona: Barcelona.

Ribas, J., Torre, I., Torrentó, J. & Jürgens, J., (2011). Efectes dels tractaments de millora de la Pinassa (*Pinus nigra*) sobre els ocells i els petits mamífers a can Dalmau (Parc Natural de Sant Llorenç del Munt i l'Obac). In: *VII Monografies de Sant Llorenç del Munt i l’Obac*: 127*.* Hernández, J., Grau, J. & Melero, J. (Eds.). Diputació de Barcelona: Barcelona.

Ribes, J., Serra, A. & Goula, M. (2004) *Catàleg dels heteròpters de Catalunya (Insecta, Hemiptera, Heteroptera)*. Barcelona: Institució Catalana d'Història Natural (ICHN).

Rieradevall, M., Bonada, N. & Prat, N. (1999). Community structure and water quality in Mediterranean streams of a Natural Park (Sant Llorenç del Munt, NE Spain). *Limnetica,* **17**, 45-56.

Rieradevall, M. & Prat, N. (1998) Estat ecològic de la Riera de la Vall d’Horta en condicions d’estiatge. Diputació de Barcelona, Barcelona.

Rivera, J. & Sáez, A. (2003). La fauna acuática introducida y su impacto sobre los anfibios y reptiles. **205**, 22-27.

Rodriguez-Lozano, P., Verkaik, I., Rieradevall, M. & Prat, N. (2015). Small but powerful: top predator local extinction affects ecosystem structure and function in an intermittent stream. *PloS one,* **10**, e0117630.

Rodríguez-Lozano, P., Verkaik, I., Rieradevall, M. & Prat, N., (2014a). Efectes potencials de la reintroducció del barb de muntanya (*Barbus meridionalis*) a la riera de la Vall d’Horta. In: *VIII Trobada d’Estudiosos de Sant Llorenç del Munt i l’Obac*: 146-155*.* Duran, C., Hernández, J., Grau, J. & Melero, J. (Eds.). Diputació de Barcelona: Barcelona.

Rodríguez-Lozano, P., Verkaik, I., Rieradevall, M. & Prat, N., (2014b). Efectes potencials de la reintroducció del barb de muntanya (Barbus meridionalis) a la riera de la Vall d’Horta in VIII Trobada d’Estudiosos de Sant Llorenç del Munt i l’Obac. In: *VIII Trobada d’Estudiosos de Sant Llorenç del Munt i l’Obac*: 146-155*.* Diputació de Barcelona (Ed.). Diputació de Barcelona: Barcelona.

Rodríguez‐Lozano, P., Verkaik, I., Maceda‐Veiga, A., Monroy, M., de Sostoa, A., Rieradevall, M. & Prat, N. (2016). A trait‐based approach reveals the feeding selectivity of a small endangered Mediterranean fish. *Ecol Evol,* **6**, 3299-3310.

Rollan, À. & Real, J. (2005) Avaluació de l’estat de les espècies cinegètiques de caça menor a l’àrea afectada per l’incendi de 2003 als termes municipals de Sant Llorenç Savall, Gallifa, Granera, Mura, Monistrol de Calders i Castellterçol. 6*.* Departament Biologia Animal, Universitat de Barcelona, Barcelona.

Rollan, À. & Real, J. (2011). Effect of wildfires and post-fire forest treatments on rabbit abundance. *Eur. J. Forest Res.,* **57**, 201-209.

Rollan, A., Real, J., Bosch, R., Tinto, A. & Hernandez-Matias, A. (2010). Modelling the risk of collision with power lines in Bonelli’s Eagle Hieraaetus fasciatus and its conservation implications. *Bird Conserv Int,* **20**, 279-294.

Rollan, À., Real, J. & Tintó, A. (2007a) Pla de gestió intergral de millora d'hàbitats per a l'àliga perdiguera (*Hieraaetus fasciatus*) a Sant Llorenç del Munt. Projecte demostratiu. 85*.* Universitat de Barcelona, Barcelona.

Rollan, À., Tintó, A. & Real, J., (2007b). Avaluació de la relació entre l’abundància relativa de conill (*Oryctolagus cuniculus*) i el tractament forestal postincendi. In: *VI Trobada d’Estudiosos de Sant Llorenç del Munt i l’Obac*: 245-248*.* Hernández, J., Grau, J. & Melero, J. (Eds.). Diputació de Barcelona: Barcelona, Soain.

Roquet, C. & Sáez, L. (2010) Estudi de la diferenciació genètica i morfològica de les poblacions d’Arenaria fontqueri al Montcau (Parc Natural de Sant Llorenç del Munt i l’Obac) i de la conservació de les codines de la zona alta d’aquesta muntanya. Diputació de Barcelona, Barcelona.

Rosalino, L.M., Loureiro, F., Macdonald, D. & Santon-Reis, M. (2005). Dietary shifts of the badger (Meles meles) in Mediterranean woodlands: an opportunistic forager with seasonal specialisms. **70**, 12-23.

Rosalino, L.M. & Santos‐Reis, M. (2009). Fruit consumption by carnivores in Mediterranean Europe. **39**, 67-78.

Rosell, C. (1999) Programa de seguiment de les poblacions de senglar (*Sus scrofa*) a Catalunya. Sant Llorenç del Munt i Serra de l’Obac temporada 1998/1999. 12*.* Minuartia. Estudis ambientals, Barcelona.

Rosell, C. & Carretero, M.Á. (1999) Programa de seguiment de les poblacions de senglar (Sus scrofa) a Catalunya. Temporada 1998/1999. 53*.* Minuartia. Estudis ambientals, Barcelona.

Rosell, C., Navàs, F. & Carol, Q. (2008) Programa de seguiment de les poblacions de senglar a Catalunya. Sant Llorenç del Munt i l'Obac. Temporada 2007/2008. 15*.* MINUARTIA, Barcelona.

Rosell, C., Navás, F. & Serra, V. (2012) Programa de seguiment de les poblacions de senglar a Catalunya. Sant Llorenç del Munt i l'Obac. Temporada 2011/2012. 20*.* MINUARTIA, Barcelona.

Rosell, C., Pasquina, À., Colomer, T. & Villero, D. (2001) Seguiment de les poblacions de carnívors del Parc Natural de Sant Llorenç del Munt i l’Obac. 2000. 72*.* Diputació de Barcelona, Barcelona.

Rosell, C. & Villero, D., (2002). Seguiment de la població de senglar (*Sus scrofa*) a Sant Llorenç del Munt i l’Obac. In: *V Trobada d’Estudiosos de Sant Llorenç del Munt i l’Obac. Monografies, 35*: 135-139*.* Xarxa de Parcs Naturals (Ed.). Diputació de Barcelona: Barcelona.

Rosich, J. & Real, J. (2016) Selecció de l’hàbitat de cria de l’astor a un ambient de muntanya mediterrània. 43*.* Universitat de Barcelona, Barcelona.

Rost, J. (2016) Desenvolupament del sistema d’indicadors de l’estat de conservació de la fauna a la Xarxa de Parcs Naturals de la Diputació de Barcelona. 211*.* Diputació de Barcelona, Àrea d'espais Naturals, Barcelona.

Rost, J., Clavero, M., Brotons, L. & Pons, P. (2012). The effect of postfire salvage logging on bird communities in Mediterranean pine forests: the benefits for declining species. *J. Appl. Ecol.,* **49**, 644-651.

Rost, J., Pons, P. & Bas, J.M. (2009). Can salvage logging affect seed dispersal by birds into burned forests? **35**, 763-768.

Sáez, L., Aymerich, P. & Blanché, C. (2010). Llibre vermell de les plantes endèmiques i amenaçades de Catalunya. 811.

Sáez, L., López, J. & Carnicero, P. (2015) Inventariació de la flora al·lòctona de caràcter invasor del Parc Natural de Sant Llorenç del Munt i Serra de l’Obac. 66*.* Diputació de Barcelona, Àrea d'espais Naturals, Barcelona.

Sáez, L., Ríos, A.I. & López, J. (2012) Cartografia i dades per a l'estudi bàsic per a la conservació de la flora de les codines del Parc Natural de Sant Llorenç del Munt i l’Obac. 28*.* Diputació de Barcelona, Àrea d'espais Naturals., Barcelona.

Salvat, A. (2013) Desenvolupament del sistema d’indicadors de l’estat de conservació de la flora i els hàbitats a la Xarxa de Parcs Naturals de la Diputació de Barcelona. 77*.* Diputació de Barcelona, Barcelona.

Salvat, A. & Monje, X. (2008a) Pla estratègic de conservació dels hàbitats i la flora de la Xarxa de Parcs Naturals de la Diputació de Barcelona (Projecte-G018). Diputació de Barcelona (Ed.). Barcelona.

Salvat, A. & Monje, X. (2008b) Pla estratègic de conservació dels hàbitats i la flora de la Xarxa de Parcs Naturals de la Diputació de Barcelona (Projecte-G018). 148*.* Diputació de Barcelona, Barcelona.

Salvat, A. & Sáez, L. (2016a) Revisió i actualització del Pla estratègic de conservació dels hàbitats i la flora de la Xarxa de Parcs Naturals de la Diputació de Barcelona: flora vascular autòctona (PECFV). 203*.* Diputació de Barcelona, Barcelona.

Salvat, A. & Sáez, L. (2016b) Revisió i actualització del Pla estratègic de conservació dels hàbitats i la flora de la Xarxa de Parcs Naturals de la Diputació de Barcelona: flora vascular autòctona (PECFV). Diputació de Barcelona (Ed.). Barcelona.

Santos, X. (2008a) Seguiment integral de la recolonització faunistica post-incendi, a la zona afectada per l’incendi del 2003, al Parc Natural de Sant Llorenç del Munt i l’Obac. Diputació de Barcelona (Ed.). Barcelona.

Santos, X. (2008b) Seguiment integral de la recolonització faunística post-incendi, a la zona afectada per l’incendi del 2003, al Parc Natural de Sant Llorenç del Munt i l’Obac. Diputació de Barcelona, Barcelona.

Santos, X., (2008c). Subprojecte Rèptils. In: Seguiment integral de la recolonització faunística post-incendi, a la zona afectada per l’incendi del 2003, al Parc Natural de Sant Llorenç del Munt i l’Obac: 171-176. Santos, X. (Ed.). Diputació de Barcelona: Barcelona.

Santos, X., Bros, V. & Mino, À. (2009). Recolonization of a burned Mediterranean area by terrestrial gastropods. **18**, 3153-3165.

Santos, X., Bros, V. & Ros, E. (2012). Contrasting responses of two xerophilous land snails to fire and natural reforestation. *Contrib Zool,* **81**, 167-180.

Santos, X., Mateos, E., Bros, V., Brotons, L., De Mas, E., Herraiz, J.A., Herrando, S., Miño, À., Olmo-Vidal, J.M. & Quesada, J. (2014). Is response to fire influenced by dietary specialization and mobility? A comparative study with multiple animal assemblages. **9**, e88224.

Santos, X., Mateos, E. & Viñolas, A. (2010). Canvis en la comunitat de coleòpters de vegetació a causa d’un incendi forestal al Parc Natural de Sant Llorenç del Munt i l’Obac. **75**, 99-118.

Santos, X. & Poquet, J.M. (2010). Ecological succession and habitat attributes affect the postfire response of a Mediterranean reptile community. *Eur. J. Forest Res.,* **56**, 895-905.

SEMICE (2008) Seguiment dels petits mamífers comuns d'Espanya (SEMICE). Museu de Granollers Ciències Naturals, Granollers, Espanya.

Serra, A., Fanlo, E., Mas, A., Mateos, E., Parra, X., Sarlé, V., Serrasolsas, I. & Socarrats, R., (1989). Primeres dades de l’estudi del sòl i dels artròpodes edàfics d’un bosc cremat. In: *I Trobada d’estudiosos de Sant Llorenç del Munt i l’Obac*: 83-91*.* Xarxa de Parcs Naturals (Ed.). Diputació de Barcelona: Barcelona.

Serra-Cobo, J. (1989). *Estudi de la biología i ecología de Miniopterus schreibersii*. PhD thesis, Universitat de Barcelona.

Serra-Cobo, J. (1998) Estudi quiroptològic dels parcs naturals del Garraf i de St. Llorenç del Munt (Projecte realitzat durant 1998). Diputació de Barcelona, Barcelona.

Serra-Cobo, J. (1999) Estudi quiropterològic dels parcs naturals del Garraf i de Sant Llorenç del Munt. 33*.* Diputació de Barcelona, Barcelona.

Serra-Cobo, J. (2000) Estudi dels quiròpters del Parc Natural de Sant Llorenç del Munt. 32*.* Diputació de Barcelona, Barcelona.

Serra-Cobo, J., (2002). Estudi quiropterològic del Parc Natural de Sant Llorenç del Munt i l’Obac. In: *V Trobada d’Estudiosos de Sant Llorenç del Munt i l’Obac. Monografies*: 147-150*.* Vila, M., Prat, N. & Rieradevall, M. (Eds.). Diputació de Barcelona: Barcelona.

Serra-Cobo, J. & Balcells, E. (1986) Mise à jour des resultats de campagnes de baguage de Miniopterus schreibersii dans le NE espagnol et le SE français. Rouen, France: Soc. Française pour l'Étude et la Protection des Mammifères.

Serra-Cobo, J. & Balcells, E., (1991). Migraciones de quirópteros en España. In: *Los murciélagos de España y Portugal*: 181-209*.* J, B. & Paz, O.d. (Eds.). Monografías del ICONA, Colección Técnica: Madrid, Esapaña.

Serra-Cobo, J., Balcells, E. & Guasch, J.F. (1989). Seasonal movements of Miniopterus schreibersii in NE Spain and SE France. **5**, 32.

Serra-Cobo, J., Bayer, X., López-Roig, M., Armengual, B. & Guasch, C. (2008) Estudi dels quiròpters del Parc Natural de Sant Llorenç del Munt i l’Obac com a base per a la seva gestió. 27*.* areaambiental.com & Diputació de Barcelona, Barcelona.

Serra-Cobo, J., López-Roig, M., Amengual, B. & Fontal, J. (2004) Estudi dels quiròpters del Parc Natural de Sant Llorenç del Munt i l’Obac com a base per a la seva gestió. 61*.* areaambiental.com & Diputació de Barcelona, Barcelona.

Serra-Cobo, J., López-Roig, M., Amengual, B. & Martínez-Rica, J.P. (2001) Estudi dels quiròpters del Parc Natural de Sant Llorenç del Munt i l’Obac. 35*.* Biputació de Barcelona, Barcelona.

Serra-Cobo, J., López-Roig, M., Amengual, B. & Martínez-Rica, J.P. (2002) Estudi dels quiròpters del Parc Natural de Sant Llorenç del Munt i l’Obac. 35*.* ecoserveis & Diputació de Barcelona, Barcelona.

Serra-Cobo, J., López-Roig, M., Amengual, B. & Martínez-Rica, J.P. (2003) Estudi dels quiròpters del Parc Natural de Sant Llorenç del Munt i l’Obac. 39*.* areambiental.com & Diputació de Barcelona, Barcelona.

Serra-Cobo, J., López-Roig, M., Marquès-Bonet, T. & Martínez-Rica, J.P. (2000). Body condition changes of *Miniopterus schreibersii* in autumn and winter. **55**, 351-360.

Serra-Cobo, J., López-Roig, M. & Sandoval, A. (2019) Conservació d’espècies prioritàries de quiròpters al Parc Natural de Sant Llorenç del Munt i l’Obac. 20*.* Areambiental de la Universitat de Barcelona, Barcelona.

Serra-Cobo, J., López-Roig, M., Torres, M., Ripoll, A., Armengual, B. & Fontal, J. (2005) Estudi dels quiròpters del Parc Natural de Sant Llorenç del Munt i l’Obac com a base per a la seva gestió. 55*.* areaambiental.com & Diputació de Barcelona, Barcelona.

Serra-Cobo, J., Sanz, V. & Martínez-Rica, J.P. (1998). Migratory movements of Miniopterus schreibersii in the north-east of Spain. **43**, 271-283.

Serrat, A., Pons, P., Puig-Gironès, R. & Stefanescu, C. (2015). Environmental factors influencing butterfly abundance after a severe wildfire in Mediterranean vegetation. **38**, 207-220.

Sesma, J.M. & Muñoz, Q., (2011). Programa de seguiment de les papallones diürnes al Parc Natural de Sant Llorenç del Munt i l’Obac. In: *VII Monografies de Sant Llorenç del Munt i l’Obac*: 75-80*.* Hernández, J., Grau, J. & Melero, J. (Eds.). Diputació de Barcelona: Barcelona.

Sesma, J.M. & Vivas, L. (2012). Nueva cita de *Zonitis fernancastroi* Pardo Alcaide, 1950 (*Coleoptera: Meloidae*) en el Parque Natural de Sant Llorenç del Munt. **1**, 75-81.

Simón, J., Estrada, M., Blanché, C. & Molero, J., (2000). Biologia de la conservació de tres espècies endèmiques del Parc Natural de Sant Llorenç del Munt i l'Obac. In: *IV Trobada d'estudiosos de Sant Llorenç del Munt i l'Obac. Monografies*: 33-43*.* Diputació de Barcelona (Ed.). Diputació de Barcelona: Barcelona.

Sostoa, A., Caiola, N. & Casals, F., (2004). A new IBI (IBICAT) for the local application of the water framework directive. In: *Proceedings of the 5th International Symposium on Ecohydraulics. Aquatic habitats: Analysis and Restoration*: 187-191*.* García de Jalón, D. & Vizcaíno Martínez, P. (Eds.). International Association of Hydraulic Engineering and Research: Madrid, España.

Sostoa, A., Caiola, N., Vinyoles, D. & Casals, F. (2002) Diagnosi de les poblacions de peixos del Parc Natural de Sant Llorenç del Munt i l’Obac. 63*.* Diputació de Barcelona, Barcelona.

Sostoa, A., Vinyoles, D., Maceda, A., Caiola, N. & Casals, F. (2006) Efectes de l’incendi del 2003 sobre les comunitats de peixos al Parc Natural de Sant Llorenç del Munt i l’Obac. 37*.* Departament de Biologia Animal, Universitat de Barcelona, Barcelona.

Sostoa, A., Vinyoles, D., Maceda, A., Caiola, N. & Casals, F., (2007). Efectes de l’incendi del 2003 sobre les comunitats de peixos al Parc Natural de Sant Llorenç del Munt i l’Obac. In: *VI Trobada d’Estudiosos de Sant Llorenç del Munt i l’Obac*: 217-221*.* Hernández, J., Grau, J. & Melero, J. (Eds.).

Stefanescu, C., Herrando, S. & Páramo, F. (2004). Butterfly species richness in the north-west Mediterranean Basin: the role of natural and human-induced factors. **31**, 905-915.

Stefanescu, C., Penuelas, J. & Filella, I. (2003). Effects of climatic change on the phenology of butterflies in the northwest Mediterranean Basin. **9**, 1494-1506.

Tauler, H., Real, J., Hernández-Matías, A., Aymerich, P., Baucells, J., Martorell, C. & Santandreu, J. (2015). Identifying key demographic parameters for the viability of a growing population of the endangered Egyptian Vulture Neophron percnopterus. . **25**, 426-439.

Tintó, A. & Real, J. (2008) Aplicació de mesures antielectrocució en línies de distribució de FECSA-ENDESA de la Serralada prelitoral de Barcelona. Valoració de les accions realitzades entre els anys 2001-2007. 71*.* Universitat de Barcelona, Barcelona.

Tintó, A., Real, J. & Mañosa, S. (2010). Predicting and Correcting Electrocution of Birds in Mediterranean Areas. **74**, 1852-1862.

Torre, I. (1998) Pla de seguiment de petits mamífers (insectívors i rosegadors) del Parc Natural de Sant Llorenç del Munt i l’Obac. 160*.* Diputació de Barcelona, Barcelona.

Torre, I. & Arrizabalaga, A., (2000). Aspectes ecològics de les comunitats de petits mamífers del Parc Natural de Sant Llorenç del Munt i l’Obac. In: *IV Trobada d’Estudiosos de Sant Llorenç del Munt i l’Obac*: 127- 131*.* Xarxa de Parcs Naturals (Ed.). Diputació de Barcelona: Barcelona.

Torre, I. & Arrizabalaga, A. (2012) Efectes dels tractaments de millora de la pinassa (*Pinus nigra*) sobre els petits mamífers a Can Dalmau (Parc de Sant Llorenç del Munt i l’Obac). Any 2011. 18*.* Diputació de Barcelona, Granollers.

Torre, I., Arrizabalaga, A., Freixas, L., Pertierra, D. & Raspall, A. (2011). Primeros resultados del programa de seguimiento de micromamíferos comunes de España (SEMICE). **23**, 81-89.

Torre, I., Díaz, M. & Arrizabalaga, A. (2014). Additive effects of climate and vegetation structure on the altitudinal distribution of greater white-toothed shrews *Crocidura russula* in a Mediterranean mountain range. *Acta Theriol,* **59**, 139-147.

Torre, I., Freixas, L. & Arrizabalaga, A. (2009a) Programa de seguiment de petits mamífers comuns al Parc de Sant Llorenç del Munt i l’Obac (Xarxa SEMICE). Any 2008. 25*.* Museu de Granollers-Ciències Naturals, Granollers.

Torre, I., Páramo, F., Carrera, D. & Dalmases, C. (2009b) Pla estratègic de conservació de la fauna de la Xarxa de Parcs Naturals de la Diputació de Barcelona (Projecte G-019). 234*.* Diputació de Barcelona, Barcelona.

Torre, I., Páramo, F., Carrera, D. & Dalmases, C. (2009c) Pla estratègic de conservació de la fauna de la Xarxa de Parcs Naturals de la Diputació de Barcelona (Projecte G-019). Diputació de Barcelona (Ed.). Barcelona.

Torre, I., Peris, A. & Tena, L. (2005). Estimating the relative abundance and temporal activity patterns of wood mice (*Apodemus sylvaticus*) by remote photography in Mediterranean post-fire habitats. *Galemys* **17**, 41-52.

Torre, I., Raspall, A., Arrizabalaga, A. & Díaz, M. (2018). SEMICE: An unbiased and powerful monitoring protocol for small mammals in the Mediterranean Region. *Mamm. Biol.*

Torre, I., Ribas, A. & Arrizabalaga, A., (2007). Efectes dels tractaments silvícoles postincendi sobre les comunitats de petits mamífers del Parc Natural de Sant Llorenç del Munt i l’Obac. In: *VI Trobada d’Estudiosos de Sant Llorenç del Munt i l’Obac*: 241-244*.* Hernández, J., Grau, J. & Melero, J. (Eds.). Diputació de Barcelona: Barcelona.

Torre, I., Ribas, A., Freixas, L. & Arrizabalaga, A., (2008a). Estudi de la distribució i abundància dels carnívors en zones cremades del Parc Natural de Sant Llorenç del Munt i l’Obac mitjançant el trampeig fotogràfic. In: *Seguiment integral de la recolonització faunística post-incendi, a la zona afectada per l’incendi del 2003, al Parc Natural de Sant Llorenç del Munt i l’Obac*: 191-212*.* Santos, X. (Ed.). Diputació de Barcelona: Barcelona.

Torre, I., Ribas, A., Freixas, L. & Arrizabalaga, A. (2008b) Seguiment de les poblacions de petits mamífers del Parc de Sant Llorenç del Munt i l’Obac (2007). In: *Granollers, Spain*: 32*.* Museu de Granollers-Ciències Naturals.

Torre, I. & Ribas, J. (2010) Efectes dels tractaments de millora de la pinassa (*Pinus nigra*) sobre els ocells i els petits mamífers a Can Dalmau (Parc de Sant Llorenç del Munt i l’Obac). 26*.* Diputació de Barcelona, Barcelona.

Torre, I. & Vilella, M. (2019) Validació i posada a punt d’una metodologia per al seguiment de mamífers carnívors al Parc natural de Sant Llorenç del Munt i l’Obac. 45*.* Museu de Granollers & Diputació de Barcelona, Granollers.

Torrentó, J., Miño, À., Agenjo, A., Muñoz, Q., Sesma, J.M. & Stefanescu, C. (2008). Actuació amb voluntaris per a la millora de l’hàbitat de la papallona *Iolana iolas* (Ochsenheimer, 1816) al Parc Natural de Sant Llorenç del Munt i l’Obac. **99**, 121-122.

Verkaik, I. & Espelta, J.M. (2006). Post-fire regeneration thinning, cone production, serotiny and regeneration age in Pinus halepensis. *Forest Ecol Manag,* **231**, 155-163.

Verkaik, I., Rieradevall, M., Cooper, S.D., Melack, J.M., Dudley, T.L. & Prat, N. (2013a). Fire as a disturbance in mediterranean climate streams. *Hydrobiologia*, 1-30.

Verkaik, I., Vila-Escalé, M., Rieradevall, M. & Prat, N. (2006) Informe de seguiment dels efectes del foc forestal de Sant Llorenç de Munt (agost 2003) sobre la xarxa de rieres de l’àrea. Característiques fisicoquímiques i comunitat de macroinvertebrats. 17*.* Departament d’Ecologia, Universitat de Barcelona, Barcelona.

Verkaik, I., Vila‐Escalé, M., Rieradevall, M. & Prat, N. (2013b). Seasonal drought plays a stronger role than wildfire in shaping macroinvertebrate communities of Mediterranean streams. *Int Rev Hydrobiol,* **98**, 271-283.

Vigo, J. (2005) *Les comunitats vegetals: descripció i classificació,* 1st edn. Barcelona: Edicions Universitat Barcelona.

Vigo, J., Carreras, J. & Ferré, A. (2005) *Manual dels hàbitats de Catalunya,* 1st edn. Barcelona: Departament de Medi Ambient i Habitatge, Generalitat de Catalunya.

Vila-Escalé, M., Prat, N. & Rieradevall, M., (2002). Estudi de la població de cranc de riu americà (*Procambarus clarkii*) i el seu efecte sobre les comunitats vegetals submergides a dos torrents del massís de Sant Llorenç del Munt i l’Obac. In: *V Trobada d’Estudiosos de Sant Llorenç del Munt i l’Obac. Monografies, 35*: 99-103*.* Xarxa de Parcs Naturals (Ed.). Diputació de Barcelona: Barcelona.

Vila-Escalé, M., Vegas, T., Verkaik, I., Rieradevall, M. & Prat, N., (2009). Canvis en l’hàbitat aquàtic a la riera de Gallifa després d’un incendi forestal. In: *VI Trobada d’Estudiosos de Sant Llorenç del Munt i l’Obac*: 199-203*.* Hernández, J., Grau, J. & Melero, J. (Eds.). Diputació de Barcelona: Barcelona.

Vila-Escalé, M., Verkaik, I., Rieradevall, M. & Prat, N., (2014). Efectes de l’incendi forestal de Sant Llorenç del Munt (2003) a la riera de Gallifa. In: *VIII Trobada d’Estudiosos de Sant Llorenç del Munt i l’Obac*: 167*.* Duran, C., Hernández, J., Grau, J. & Melero, J. (Eds.). Diputació de Barcelona: Barcelona.

Vila-Escalé, M., Verkaik, I., Vegas, T., Rieradevall, M. & Prat, N., (2007). Evolució de la comunitat de macroinvertebrats en un riu mediterrani després d’un incendi forestal. In: *VI Trobada d’Estudiosos de Sant Llorenç del Munt i l’Obac*: 205-209*.* Diputació de Barcelona: Barcelona.

Vilatersana, R. (2002) Base de dades florística del Parc de Sant Llorenç del Munt i l’Obac. Diputació de Barcelona (Ed.). Barcelona.

Villero, D. (2001) Punts d’interès per a la reproducció d'amfibis al Parc Natural de Sant Llorenç del Munt i l'Obac. 57*.* Diputació de Barcelona, Barcelona.

Villero, D. (2002) Fonaments del seguiment de les poblacions d’amfibis al Parc Natural de Sant Llorenç del Munt i l'Obac. Diputació de Barcelona, Barcelona.

Villero, D. (2003a) Actualització de la base de dades de citacions d’artròpodes del Parc Natural de Sant Llorenç del Munt i l’Obac. Diputació de Barcelona (Ed.). Barcelona.

Villero, D. (2003b) Guia metodològica per al seguiment de les poblacions d’amfibis a Sant Llorenç del Munt i l’Obac. 55*.* Diputació de Barcelona, Barcelona.

Villero, D. (2019) Canvis en la comunitat d’amfibis en els darrers 20 anys al Parc Natural de Sant Llorenç del Munt i l’Obac. 18*.* Diputació de Barcelona, Barcelona.

Villero, D., Llorente, G.A. & Montori, A., (2007). El tritó verd (*Triturus marmoratus, Urodela*) a Sant Llorenç del Munt i l’Obac. In: *VI Trobada d'Estudiosos de Sant Llorenç del Munt i l'Obac.* Hernández, J., Grau, J. & Melero, J. (Eds.). Diputació de Barcelona: Barcelona.

Villero, D., Montori, A. & Llorente, G.A. (2006). Alimentación de los adultos de *Triturus marmoratus* (*Urodela, Salamandridae*) durante el período reproductor en Sant Llorenç del Munt, Barcelona. **20**, 57-70.

Viñolas, A., Trócoli, S. & Piera , E. (2017) Proposta d’estudi dels coleòpters detritívors i saproxílics del Parc Natural de Sant Llorenç del Munt i l’Obac. 4*.* Museu de Ciències Naturals de Barcelona, Barcelona.

Vives, E. & García, A., (1989). Escarabèids copròfags del massís de Sant Llorenç del Munt i serra de l’Obac. In: *I Trobada d’estudiosos de Sant Llorenç del Munt i l’Obac*: 61-64*.* Xarxa de Parcs Naturals (Ed.). Diputació ed Barcelona: Barcelona.

Vives, E. & Vives, J., (1989). La fauna cavernícola de Sant Llorenç del Munt i serra de l’Obac. In: *I Trobada d’estudiosos de Sant Llorenç del Munt i l’Obac.* Xarxa de Parcs Naturals (Ed.). Diputació de Barcelona: Barcelona.

Vives, E. & Vives, J. (1993). Nota sobre caràbids (*Coleoptera*) de Catalunya. 1ª Nota. **VIII**, 29-36.

Zozaya, E.L., Peris, A., Bros, V., Guinart, D., Bosch, R., Tintó, A. & Real, J., (2007). Determinació de l’àrea de campeig i ritme d’activitat de l’àliga cuabarrada (*Hieraaetus fasciatus*) al Parc Natural de Sant Llorenç del Munt i l’Obac. In: *VI Trobada d’Estudiosos de Sant Llorenç del Munt i l’Obac*: 117-120*.* Hernández, J., Grau, J. & Melero, J. (Eds.). Diputació de Barcelona: Barcelona.
