# Supplementary material for: A comprehensive but practical methodology for selecting biological indicators for long-term monitoring
Source: PLoS One. 2022 Mar 15;17(3):e0265246. doi: 10.1371/journal.pone.0265246 (PMC8923439; doi:10.1371/journal.pone.0265246)
Supplement: S1 Table — List of the Individual species indicators of the Natural Park of Sant Llorenç del Munt i l’Obac following the criterion of achieving a value of the Relevance index. The list includes the taxon community or aggrupation (bold font), the accepted name of the species, the Relevance index for each species, the average for the community or aggrupation (bold font), and the four subsections to extract this index: the degree of threat (scoring ranges from 0 to 3), the monitoring programs set (0 to 1), the ecological interest (0 to 2) and the expert or taxon specialist criterion (0 to 2). (DOCX) [file pone.0265246.s002.docx]

| S1 Table. List and selection values of species List of the Individual species indicators of the Natural Park of Sant Llorenç del Munt i l’Obac following the criterion of achieving a value of the Relevance index. The list includes the taxon community or aggrupation (bold font), the accepted name of the species, the Relevance index for each species, the average for the community or aggrupation (bold font) and the four subsections to extract this index: the degree of threat (scoring ranges from 0 to 3), the ecological interest (0 to 3) and the expert or taxon specialist criterion (0 to 2). | | | | |
| --- | --- | --- | --- | --- |
| **Species/Community** | **Relevance index** | **Degree of threat** | **Ecological interest** | **Expert criterion** |
| **Fungi** | **4.00** | **0.00** | **2.00** | **2.00** |
| **Lichens** | **4.01** | **0.01** | **2.00** | **2.00** |
| **Bryophyta** | **4.01** | **0.01** | **2.00** | **2.00** |
| **Threatened flora** | **7.00** | **2.30** | **2.70** | **2.00** |
| *Dactylorhiza insularis (*Sommier*)* | 7 | 2 | 3 | 2 |
| *Campanula speciosa* subsp*. affinis (*Schult*)* | 6 | 2 | 2 | 2 |
| *Arenaria conimbricensis* Brot*.* subsp*. conimbricensis* | 6 | 2 | 2 | 2 |
| *Arenaria fontqueri (*Cardona & J. M. Monts.) | 7 | 2 | 3 | 2 |
| *Spiranthes aestivalis (*Poir*)* | 7 | 2 | 3 | 2 |
| *Silene viridiflora (*L*.)* | 7 | 2 | 3 | 2 |
| *Myricaria germanica (*L*.)* | 7 | 2 | 3 | 2 |
| *Erodium glandulosum (*Cav.) Willd. | 7 | 3 | 2 | 2 |
| *Delphinium fissum* subsp*. bolosii (*Blanché & Molero) J. Vigo | 8 | 3 | 3 | 2 |
| *Saxifraga callosa* subsp*. catalaunica (*Boiss. & Reuter) Webb | 8 | 3 | 3 | 2 |
| **Pteridophyte** | **4.00** | **0.00** | **2.00** | **2.00** |
| *Equisetum telmateia (*Ehrh*)* | 4 | 0 | 2 | 2 |
| *Selaginella denticulata (*L*.)* | 4 | 0 | 2 | 2 |
| *Asplenium onopteris (*L*.)* | 4 | 0 | 2 | 2 |
| *Asplenium scolopendrium* subsp*. scolopendrium* | 4 | 0 | 2 | 2 |
| *Asplenium trichomanes (*L*.) subsp. quadrivalens* | 4 | 0 | 2 | 2 |
| *Dryopteris filix-mas (*L*.)* | 4 | 0 | 2 | 2 |
| *Polystichum aculeatum (*L*.)* | 4 | 0 | 2 | 2 |
| *Adiantum capillus-veneris (*L.*)* | 4 | 0 | 2 | 2 |
| *Anogramma leptophylla (*L.) Link | 4 | 0 | 2 | 2 |
| *Cheilanthes acrostica (*Balb*)* | 4 | 0 | 2 | 2 |
| *Cheilanthes maderensis (*Lowe*)* | 4 | 0 | 2 | 2 |
| *Cheilanthes pteridioides (*Reich*)* | 4 | 0 | 2 | 2 |
| *Paragymnopteris marantae (*L.) K.H. Shing | 4 | 0 | 2 | 2 |
| **Vascular plants** | **4.12** | **0.22** | **2.02** | **1.88** |
| *Allium scorodoprasum (*L*.)* | 4 | 0 | 2 | 2 |
| *Narcissus assoanus (*Dufour*)* | 4 | 0 | 2 | 2 |
| *Narcissus dubius (*Gouan*)* | 5 | 1 | 2 | 2 |
| *Orchis provincialis (*Balb*)* | 4 | 0 | 2 | 2 |
| *Serapias vomeracea (*Burmf*)* | 5 | 1 | 2 | 2 |
| *Fritillaria pyrenaica* subsp*. boissieri (*Costa*)* | 4 | 0 | 2 | 2 |
| *Apera interrupta (*L*.)* | 4 | 1 | 2 | 1 |
| *Eragrostis minor (*Host*)* | 4 | 0 | 2 | 2 |
| *Festuca rubra (*L*.)* | 4 | 0 | 2 | 2 |
| *Carum carvi (*L.) | 4 | 0 | 2 | 2 |
| *Chaerophyllum temulum (*L*.)* | 4 | 0 | 2 | 2 |
| **Species/Community** | **Relevance index** | **Degree of threat** | **Ecological interest** | **Expert criterion** |
| *Orlaya daucoides (*L*.)* | 4 | 0 | 2 | 2 |
| *Ilex aquifolium (*L*.)* | 5 | 1 | 2 | 2 |
| *Aster willkommii* subsp*. catalaunicus (*Willk & Costa*)* | 4 | 0 | 2 | 2 |
| *Centaurea hanrii (*Jord*)* | 4 | 0 | 2 | 2 |
| *Hieracium patens* subsp*. pseuderiophorum (*Loret & Timb-Lagr) | 4 | 0 | 2 | 2 |
| *Oligochaeta* sp*.* | 4 | 0 | 3 | 1 |
| *Cardamine heptaphylla (*L*.)* | 4 | 0 | 2 | 2 |
| *Clypeola jonthlaspi (*L*.)* | 4 | 1 | 2 | 1 |
| *Erysimum incanum (*Kunze*)* | 4 | 0 | 2 | 2 |
| *Buxus sempervirens (L.)* | 4 | 0 | 2 | 2 |
| *Valerianella echinata (*L*.)* | 4 | 0 | 2 | 2 |
| *Arbutus unedo (*L*.)* | 4 | 0 | 2 | 2 |
| *Primula veris (*L.) | 4 | 0 | 2 | 2 |
| *Alnus glutinosa (*L.) | 4 | 0 | 2 | 2 |
| *Quercus ilex (*L.) | 4 | 0 | 2 | 2 |
| *Galium verrucosum (*Huds*)* | 4 | 0 | 2 | 2 |
| *Lathraea squamaria (*L.*)* | 4 | 1 | 2 | 1 |
| *Rosmarinus officinalis (*L*.)* | 4 | 0 | 2 | 2 |
| *Teucrium polium (*L.*)* | 4 | 0 | 2 | 2 |
| *Thymus vulgaris (*L*.)* | 4 | 0 | 2 | 2 |
| *Orobanche artemisiae-campestris (*Gaudin*)* | 5 | 2 | 2 | 1 |
| *Orobanche lavandulacea* Rchb. subsp*. lavandulacea* | 4 | 1 | 2 | 1 |
| *Viola willkommii* (R de Roemer) | 4 | 0 | 2 | 2 |
| *Helleborus foetidus* (L.) | 4 | 0 | 2 | 2 |
| *Rhamnus alaternus* (L.) | 5 | 0 | 2 | 3 |
| *Pistacia lentiscus* (L.) | 4 | 0 | 2 | 2 |
| *Juniperus oxycedrus* (L.) subsp*. oxycedrus* | 4 | 0 | 2 | 2 |
| *Pinus halepensis* (Mill) | 4 | 0 | 2 | 2 |
| *Pinus nigra* subsp*. salzmannii* (Dunal) | 4 | 0 | 2 | 2 |
| *Pinus sylvestris* (L.) | 4 | 0 | 2 | 2 |
| **Alien flora** | **5.20** | **2.00** | **1.60** | **1.60** |
| *Arundo donax (Linnaeus 1758)* | 5 | 2 | 2 | 1 |
| *Cortaderia selloana (Schult & Schultf) Asch & Graebn* | 4 | 2 | 1 | 1 |
| *Senecio pterophorus (DC.)* | 5 | 2 | 1 | 2 |
| *Robinia pseudoacacia (Linnaeus 1758)* | 6 | 2 | 2 | 2 |
| *Ailanthus altissima (Miller) Swingle* | 6 | 2 | 2 | 2 |
| **Freshwater macroinvertebrates** | **4.11** | **0.02** | **2.98** | **1.11** |
| **Odonata** | **4.27** | **0.23** | **2.88** | **1.15** |
| *Aeshna cyanea (Müller 1764)* | 5 | 0 | 3 | 2 |
| *Aeshna mixta (Latreille 1805)* | 4 | 0 | 3 | 1 |
| *Boyeria irene (Fonscolombe 1838)* | 5 | 0 | 3 | 2 |
| *Calopteryx haemorrhoidalis (Vander Linden 1825)* | 5 | 1 | 3 | 1 |
| *Calopteryx virgo subsp. meridionalis (Selys 1853)* | 4 | 0 | 3 | 1 |
| *Coenagrion caerulescens (Fonscolombe 1838)* | 4 | 1 | 2 | 1 |
| *Coenagrion mercuriale (Charpentier 1840)* | 6 | 3 | 2 | 1 |
| *Ischnura elegans (Vander Linden 1820)* | 4 | 0 | 3 | 1 |
| *Ischnura graellsii (Rambur 1842)* | 4 | 0 | 3 | 1 |
| *Ischnura pumilio (Charpentier 1825)* | 4 | 0 | 3 | 1 |
| *Nehalennia sp.* | 4 | 0 | 3 | 1 |
| *Pyrrhosoma nymphula (Sulzer 1776)* | 5 | 0 | 3 | 2 |
| *Cordulegaster boltonii subsp. boltoni (Donovan 1807)* | 4 | 0 | 3 | 1 |
| *Oxygastra curtisii (Dale 1834)* | 4 | 1 | 2 | 1 |
| **Species/Community** | **Relevance index** | **Degree of threat** | **Ecological interest** | **Expert criterion** |
| *Gomphus graslinii (Rambur 1842)* | 4 | 0 | 3 | 1 |
| *Gomphus pulchellus (Sélys 1840)* | 5 | 0 | 3 | 2 |
| *Onychogomphus forcipatus (Linnaeus 1758)* | 4 | 0 | 3 | 1 |
| *Lestes viridis (Vander Linden 1825)* | 4 | 0 | 3 | 1 |
| *Leucorrhinia pectoralis (Charpentier 1825)* | 4 | 0 | 3 | 1 |
| *Orthetrum brunneum (Fonscolombe 1838)* | 4 | 0 | 3 | 1 |
| *Orthetrum cancellatum (Linneaus 1758)* | 4 | 0 | 3 | 1 |
| *Orthetrum coerulescens (Fabricus 1798)* | 4 | 0 | 3 | 1 |
| *Sympetrum fonscolombii (Sélys 1840)* | 4 | 0 | 3 | 1 |
| *Sympetrum sinaiticum (Dumont 1977)* | 4 | 0 | 3 | 1 |
| *Sympetrum striolatum (Charpentier 1825)* | 4 | 0 | 3 | 1 |
| *Platycnemis latipes (Rambur 1842)* | 4 | 0 | 3 | 1 |
| ***Gastropoda*** | **4.42** | **0.42** | **2.00** | **2.00** |
| *Abida secale subsp. bofilli (Fagot, 1884)* | 5 | 1 | 2 | 2 |
| *Granopupa granum (Draparnaud, 1801)* | 4 | 0 | 2 | 2 |
| *Jaminia quadridens (Müller, 1774)* | 4 | 0 | 2 | 2 |
| *Euconulus (Euconulus) fulvus (Müller 1774)* | 4 | 0 | 2 | 2 |
| *Montserratina bofilliana (Fagot, 1884)* | 6 | 2 | 2 | 2 |
| *Xerocrassa montserratensis (Hidalgo, 1870)* | 6 | 2 | 2 | 2 |
| *Xerocrassa penchinati (Bourguignat, 1868)* | 4 | 0 | 2 | 2 |
| *Oxychilus (Ortizius) courquini (Bourguignat, 1870)* | 4 | 0 | 2 | 2 |
| *Vitrea contracta (Westerlund, 1871)* | 4 | 0 | 2 | 2 |
| *Punctum (Punctum) pygmaeum (Draparnaud, 1801)* | 4 | 0 | 2 | 2 |
| *Acanthinula aculeata (Müller, 1774)* | 4 | 0 | 2 | 2 |
| *Truncatellina callicratis (Scacchi, 1833)* | 4 | 0 | 2 | 2 |
| **Decomposers & Saproxylics** | **4.12** | **0.16** | **2.00** | **1.95** |
| *Oligochaeta* | 4 | 0 | 2 | 2 |
| *Glomeris marginata (Villers 1789)* | 4 | 0 | 2 | 2 |
| *Microhoria fasciata (Chevrolat, 1834)* | 4 | 0 | 2 | 2 |
| *Pogonocherus caroli (Mulsant, 1862)* | 4 | 0 | 2 | 2 |
| *Albana m-griseum (Mulsant, 1846)* | 4 | 0 | 2 | 2 |
| *Aromia moschata (Linnaeus, 1758)* | 4 | 0 | 2 | 2 |
| *Cerambyx cerdo subsp. mirbeckii (Lucas, 1842)* | 5 | 2 | 2 | 1 |
| *Chlorophorus ruficornis (Olivier, 1790)* | 4 | 0 | 2 | 2 |
| *Chlorophorus varius (Müller, 1766)* | 4 | 0 | 2 | 2 |
| *Lucasianus levaillantii (Lucas, 1849)* | 4 | 0 | 2 | 2 |
| *Necydalis ulmi (Chevrolat, 1838)* | 5 | 1 | 2 | 2 |
| *Parmena meregallii (Sama, 1984)* | 4 | 0 | 2 | 2 |
| *Pedostrangalia revestita (Linnaeus, 1767)* | 4 | 1 | 2 | 1 |
| *Pseudosphegesthes cinerea (Castelnau & Gory, 1841)* | 4 | 0 | 2 | 2 |
| *Purpuricenus ferrugineus (Fairmaire, 1851)* | 4 | 0 | 2 | 2 |
| *Purpuricenus globulicollis (Dejean, 1839)* | 4 | 0 | 2 | 2 |
| *Pyrrhidium sanguineum (Linnaeus, 1758)* | 4 | 0 | 2 | 2 |
| *Saperda (Lopezcolonia) punctata (Linnaeus, 1767)* | 5 | 1 | 2 | 2 |
| *Stenopterus ater (Linnaeus, 1767)* | 4 | 0 | 2 | 2 |
| *Stenurella (Nigrostenurella) nigra (Linnaeus, 1758)* | 4 | 0 | 2 | 2 |
| *Stenurella (Priscostenurella) bifasciata (Müller, 1776)* | 4 | 0 | 2 | 2 |
| *Stictoleptura cordigera (Fueßlins, 1775)* | 4 | 0 | 2 | 2 |
| *Stictoleptura fontenayi (Mulsant, 1839)* | 4 | 0 | 2 | 2 |
| **Species/Community** | **Relevance index** | **Degree of threat** | **Ecological interest** | **Expert criterion** |
| *Stictoleptura trisignata (Fairmaire, 1852)* | 5 | 1 | 2 | 2 |
| *Vesperus aragonicus (Baraud, 1964)* | 4 | 0 | 2 | 2 |
| *Vesperus xatarti (Mulsant, 1839)* | 4 | 0 | 2 | 2 |
| *Cetonia carthami subsp. aurataeformis (Curti, 1913)* | 4 | 0 | 2 | 2 |
| *Protaetia (Netocia) morio (Fabricius, 1781)* | 4 | 0 | 2 | 2 |
| *Protaetia (Netocia) oblonga (Gory & Percheron, 1833)* | 4 | 0 | 2 | 2 |
| *Clambus hayekae (Endrody-Younga, 1960)* | 4 | 0 | 2 | 2 |
| *Arthrolips convexiuscula (Motschulsky, 1849)* | 4 | 0 | 2 | 2 |
| *Sericoderus pecirkanus (Reitter, 1908)* | 4 | 0 | 2 | 2 |
| *Atomaria (Atomaria) analis (Erichson, 1846)* | 4 | 0 | 2 | 2 |
| *Baridius quadraticollis (Boheman, 1844)* | 4 | 0 | 2 | 2 |
| *Dienerella (Cartoderema) clathrata (Mannrheim, 1844)* | 4 | 0 | 2 | 2 |
| *Colon (Colon) cloueti (Guillebeau, 1896)* | 4 | 0 | 2 | 2 |
| *Colon (Myloechus) emarginatum* (Rosenhauer, 1856) | 4 | 0 | 2 | 2 |
| *Colon (Myloechus) griseum* (Czwalina, 1881) | 4 | 0 | 2 | 2 |
| *Lucanus cervus* (Linnaeus, 1758) | 5 | 1 | 2 | 2 |
| *Lucanus pontbrianti (Mulsant, 1839)* | 4 | 0 | 2 | 2 |
| *Enicopus (Parahenicopus) vittatus (Kiesenwetter, 1859)* | 4 | 0 | 2 | 2 |
| *Nacerdes (Xanthochroa) raymondi (Mulsant & Godart, 1860)* | 4 | 0 | 2 | 2 |
| *Isomira hispanica (Kiersenwetter, 1870)* | 4 | 0 | 2 | 2 |
| **Formicidae** | **4.14** | **0.14** | **2.00** | **2.00** |
| *Aphaenogaster gibbosa (Latreille, 1798)* | 4 | 0 | 2 | 2 |
| *Aphaenogaster senilis (Mayr, 1853)* | 4 | 0 | 2 | 2 |
| *Camponotus (Myrmentoma) piceus (Leach, 1825)* | 4 | 0 | 2 | 2 |
| *Camponotus (Myrmosericus) cruentatus (Latreille, 1802)* | 4 | 0 | 2 | 2 |
| *Crematogaster auberti (Emery 1869)* | 4 | 0 | 2 | 2 |
| *Crematogaster scutellaris (Olivier, 1792)* | 4 | 0 | 2 | 2 |
| *Crematogaster sordidula (Nylander, 1849)* | 4 | 0 | 2 | 2 |
| *Goniomma blanchi (Andre, 1881)* | 4 | 0 | 2 | 2 |
| *Leptothorax (Temnothorax) kutteri (Ward et al. 2014)* | 6 | 2 | 2 | 2 |
| *Leptothorax (Temnothorax) niger (Forel 1894)* | 4 | 0 | 2 | 2 |
| *Leptothorax (Temnothorax) racovitzai (Bondroit 1918)* | 5 | 1 | 2 | 2 |
| *Leptothorax (Temnothorax) recedens (Nylander 1856)* | 4 | 0 | 2 | 2 |
| *Messor barbarus (Linnaeus 1767)* | 4 | 0 | 2 | 2 |
| *Messor bouvieri (Bondroit 1918)* | 4 | 0 | 2 | 2 |
| *Messor capitatus (Latreille 1798)* | 4 | 0 | 2 | 2 |
| *Messor structor (Latreille 1798)* | 4 | 0 | 2 | 2 |
| *Pheidole pallidula (Nylander 1849)* | 4 | 0 | 2 | 2 |
| *Solenopsis latro (Forel 1894)* | 4 | 0 | 2 | 2 |
| *Tapinoma nigerrimum (Nylander 1856)* | 4 | 0 | 2 | 2 |
| *Tetramorium caespitum (Linnaeus 1758)* | 4 | 0 | 2 | 2 |
| *Tetramorium forte (Forel 1904)* | 4 | 0 | 2 | 2 |
| *Tetramorium semilaeve (André 1883)* | 4 | 0 | 2 | 2 |
| **Orthoptera** | **4.13** | **0.13** | **2.00** | **2.00** |
| **Species/Community** | **Relevance index** | **Degree of threat** | **Ecological interest** | **Expert criterion** |
| *Acrotylus insubricus (Scopoli 1786)* | 4 | 0 | 2 | 2 |
| *Aiolopus strepens (Latreille 1804)* | 4 | 0 | 2 | 2 |
| *Brachycrotaphus tryxalicerus (Fischer 1853)* | 5 | 1 | 2 | 2 |
| *Calliptamus barbarus (Costa 1836)* | 4 | 0 | 2 | 2 |
| *Chorthippus (Glyptobothrus) jacobsi (Harz 1975)* | 4 | 0 | 2 | 2 |
| *Chorthippus (Glyptobothrus) vagans (Eversmann 1848)* | 4 | 0 | 2 | 2 |
| *Euchorthippus chopardi (Descamps 1968)* | 4 | 0 | 2 | 2 |
| *Locusta migratoria (Linnaeus 1758)* | 4 | 0 | 2 | 2 |
| *Oedipoda caerulescens (Linnaeus 1758)* | 4 | 0 | 2 | 2 |
| *Oedipoda coerulea (Saussure 1884)* | 4 | 0 | 2 | 2 |
| *Omocestus (Dreuxius) minutissimus (Brullé 1832)* | 4 | 0 | 2 | 2 |
| *Ramburiella (Ramburiella) hispanica (Rambur 1838)* | 4 | 0 | 2 | 2 |
| *Sphingonotus (Sphingonotus) caerulans (Linnaeus 1767)* | 4 | 0 | 2 | 2 |
| *Parasteropleurus perezii (Bolívar 1877)* | 4 | 0 | 2 | 2 |
| *Anacridium aegyptium (Linnaeus 1764)* | 4 | 0 | 2 | 2 |
| *Eugryllodes pipiens (Dufour 1820)* | 4 | 0 | 2 | 2 |
| *Gryllomorpha (Gryllomorphella) uclensis (Pantel 1890)* | 4 | 0 | 2 | 2 |
| *Gryllotalpa gryllotalpa (Linnaeus 1758)* | 4 | 0 | 2 | 2 |
| *Pyrgomorpha (Pyrgomorpha) conica (Olivier 1791)* | 4 | 0 | 2 | 2 |
| *Barbitistes fischeri (Yersin 1854)* | 4 | 0 | 2 | 2 |
| *Conocephalus (Anisoptera) fuscus (Fabricius 1793)* | 4 | 0 | 2 | 2 |
| *Decticus albifrons (Fabricius 1775)* | 4 | 0 | 2 | 2 |
| *Phaneroptera (Phaneroptera) nana (Fieber 1853)* | 4 | 0 | 2 | 2 |
| *Ruspolia nitidula (Scopoli 1786)* | 4 | 0 | 2 | 2 |
| *Saga pedo (Pallas 1771)* | 7 | 3 | 2 | 2 |
| *Tessellana tessellata (Charpentier 1825)* | 4 | 0 | 2 | 2 |
| *Tettigonia viridissima (Linnaeus 1758)* | 4 | 0 | 2 | 2 |
| *Thyreonotus corsicus (Rambur 1838)* | 4 | 0 | 2 | 2 |
| *Tylopsis lilifolia (Fabricius 1793)* | 4 | 0 | 2 | 2 |
| *Yersinella raymondii (Yersin 1860)* | 4 | 0 | 2 | 2 |
| **Lepidoptera** | **5.39** | **0.97** | **2.83** | **1.58** |
| *Artimelia latreillei (Godart 1823)* | 5 | 2 | 2 | 1 |
| *Carcharodus boeticus (Rambur 1839)* | 5 | 1 | 3 | 1 |
| *Carcharodus lavatherae (Esper 1783)* | 5 | 1 | 3 | 1 |
| *Erynnis tages (Linnaeus 1758)* | 6 | 1 | 3 | 2 |
| *Thymelicus acteon (Rottemburg 1775)* | 6 | 2 | 3 | 1 |
| *Thymelicus lineola (Ochsenheimer 1808)* | 5 | 1 | 3 | 1 |
| *Thymelicus sylvestris (Poda 1761)* | 5 | 1 | 3 | 1 |
| *Callophrys avis (Chapman 1909)* | 6 | 1 | 3 | 2 |
| *Cupido argiades (Pallas 1771)* | 4 | 1 | 2 | 1 |
| *Glaucopsyche melanops (Boisduval 1829)* | 6 | 1 | 3 | 2 |
| *Iolana iolas (Ochsenheimer 1816)* | 7 | 2 | 3 | 2 |
| *Laeosopis roboris (Esper 1790)* | 6 | 1 | 3 | 2 |
| *Maculinea arion (Linnaeus 1758)* | 5 | 2 | 2 | 1 |
| *Polyommatus (Lysandra) hispana (Herrich-Schäffer 1852)* | 6 | 1 | 3 | 2 |
| *Polyommatus (Polyommatus) icarus (Rottemburg 1775)* | 5 | 0 | 3 | 2 |
| *Neozephyrus quercus (Linnaeus 1758)* | 5 | 0 | 3 | 2 |
| *Satyrium acaciae (Fabricius 1787)* | 5 | 1 | 3 | 1 |
| *Satyrium esculi (Hübner 1804)* | 6 | 1 | 3 | 2 |
| **Species/Community** | **Relevance index** | **Degree of threat** | **Ecological interest** | **Expert criterion** |
| *Satyrium spini (Denis & Schiffermüller 1775)* | 5 | 0 | 3 | 2 |
| *Tomares ballus (Fabricius 1787)* | 7 | 2 | 3 | 2 |
| *Coenonympha dorus (Esper 1782)* | 5 | 1 | 3 | 1 |
| *Hipparchia (Hipparchia) fagi (Scopoli 1763)* | 4 | 2 | 2 | 0 |
| *Lasiommata megera (Linnaeus 1767)* | 5 | 0 | 3 | 2 |
| *Maniola jurtina (Linnaeus 1758)* | 5 | 0 | 3 | 2 |
| *Melanargia lachesis (Hübner 1790)* | 6 | 1 | 3 | 2 |
| *Pyronia (Idata) cecilia (Vallantin 1894)* | 5 | 0 | 3 | 2 |
| *Pyronia (Pyronia) tithonus (Linnaeus 1767)* | 4 | 0 | 3 | 1 |
| *Euphydryas (Eurodryas) aurinia (Rottemburg 1775)* | 8 | 3 | 3 | 2 |
| *Melitaea deione (Geyer 1832)* | 6 | 1 | 3 | 2 |
| *Melitaea didyma (Esper 1780)* | 4 | 1 | 2 | 1 |
| *Melitaea parthenoides (Keferstein 1851)* | 5 | 0 | 3 | 2 |
| *Leptidea sinapis (Linnaeus 1758)* | 5 | 0 | 3 | 2 |
| *Pieris rapae (Linnaeus 1758)* | 5 | 0 | 3 | 2 |
| *Pontia daplidice (Linnaeus 1758)* | 5 | 0 | 3 | 2 |
| *Hamearis lucina (Linnaeus 1758)* | 5 | 1 | 3 | 1 |
| *Graellsia isabellae (Graëlls 1849)* | 7 | 3 | 2 | 2 |
| **Other invertebrates of special interest** | **4.40** | **0.80** | **1.90** | **1.70** |
| *Roncus caballeroi (Lagar, 1974)* | 4 | 2 | 1 | 1 |
| *Timarcha (Timarchostoma) monserratensis (Bechyne, 1962)* | 6 | 2 | 2 | 2 |
| *Lampyris iberica (Geisthardt et al., 2008)* | 4 | 0 | 2 | 2 |
| *Nyctophila reichii Jaquelin du Val, 1859* | 4 | 0 | 2 | 2 |
| *Mylabris (Mylabris) quadripunctata (Linnaeus, 1767)* | 4 | 0 | 2 | 2 |
| *Abax (Abax) pyrenaeus (Dejean, 1828)* | 4 | 0 | 2 | 2 |
| *Aquarius najas (De Geer, 1773)* | 4 | 0 | 2 | 2 |
| *Apis mellifera (Linnaeus, 1758)* | 4 | 0 | 2 | 2 |
| *Austropotamobius pallipes (Lereboullet, 1858)* | 6 | 3 | 2 | 1 |
| *Stenasellus virei (Dollfus, 1898)* | 4 | 1 | 2 | 1 |
| **Plague species** | **4.14** | **0.00** | **2.14** | **2.00** |
| *Ips sexdentatus (Börner, 1776)* | 4 | 0 | 2 | 2 |
| *Ips sp.* | 4 | 0 | 2 | 2 |
| *Tomicus piniperda (Linnaeus, 1758)* | 4 | 0 | 2 | 2 |
| *Kermes vermilio (Planchon, 1864)* | 4 | 0 | 2 | 2 |
| *Lymantria dispar (Linnaeus 1758)* | 4 | 0 | 2 | 2 |
| *Thaumetopoea pityocampa (Schiffermüller, 1776)* | 5 | 0 | 3 | 2 |
| *Tortrix sp.* | 4 | 0 | 2 | 2 |
| **Alien invertebrates** | **4.60** | **1.20** | **2.20** | **1.20** |
| *Potamopyrgus antipodarum (Gray 1843)* | 5 | 2 | 2 | 1 |
| *Paralaoma servilis (Shuttleworth, 1852)* | 4 | 0 | 2 | 2 |
| *Lasius (Lasius) neglectus (Van Loon et al. 1990)* | 4 | 1 | 2 | 1 |
| *Linepithema humile (Mayr 1868)* | 4 | 1 | 2 | 1 |
| *Procambarus (Scapulicambarus) clarkii (Girard, 1852)* | 6 | 2 | 3 | 1 |
| **Freshwater fishes** | **8.00** | **3.00** | **3.00** | **2.00** |
| *Barbus haasi (Mertens, 1925)* | 8 | 3 | 3 | 2 |
| *Barbus meridionalis (Risso, 1827)* | 8 | 3 | 3 | 2 |
| *Squalius laietanus (Doadrio Kottelat & de Sostoa 2007)* | 8 | 3 | 3 | 2 |
| **Amphibian** | **5.71** | **1.43** | **3.00** | **1.29** |
| *Alytes obstetricans (Laurenti 1768)* | 6 | 2 | 3 | 1 |
| *Bufo spinosus (Linnaeus 1758)* | 4 | 0 | 3 | 1 |
| **Species/Community** | **Relevance index** | **Degree of threat** | **Ecological interest** | **Expert criterion** |
| *Epidalea calamita (Laurenti 1768)* | 6 | 2 | 3 | 1 |
| *Hyla meridionalis (Boettger 1874)* | 6 | 2 | 3 | 1 |
| *Pelophylax perezi (López-Seoane 1885)* | 7 | 2 | 3 | 2 |
| *Salamandra salamandra (Linnaeus 1758)* | 6 | 1 | 3 | 2 |
| *Triturus marmoratus (Latreille 1800)* | 5 | 1 | 3 | 1 |
| **Reptiles** | **5.50** | **2.00** | **2.00** | **1.50** |
| *Rhinechis scalaris (Duméril & Bibron, 1854)* | 4 | 1 | 2 | 1 |
| *Vipera latastei (Bosca 1878)* | 6 | 2 | 2 | 2 |
| *Podarcis liolepis (Boulenger 1905)* | 6 | 2 | 2 | 2 |
| *Psammodromus algirus (Linnaeus 1758)* | 5 | 2 | 2 | 1 |
| *Timon lepidus (Daudin 1802)* | 6 | 2 | 2 | 2 |
| *Trachemys scripta subsp. elegans (Wied 1838)* | 6 | 3 | 2 | 1 |
| *Mauremys leprosa (Schweigger 1812)* | 6 | 3 | 2 | 1 |
| **Raptors** | **6.00** | **1.77** | **2.92** | **1.31** |
| *Accipiter gentilis (Linnaeus 1758)* | 6 | 1 | 3 | 2 |
| *Accipiter nisus (Linnaeus, 1758)* | 5 | 1 | 3 | 1 |
| *Aquila fasciata (Vieillot 1822)* | 8 | 3 | 3 | 2 |
| *Buteo buteo (Linnaeus 1758)* | 4 | 0 | 3 | 1 |
| *Circaetus gallicus (Gmelin, 1788)* | 5 | 2 | 3 | 0 |
| *Hieraaetus pennatus (Gmelin, 1788)* | 4 | 2 | 3 | 0 |
| *Neophron percnopterus (Linnaeus 1758)* | 8 | 3 | 3 | 2 |
| *Falco peregrinus (Tunstall 1771)* | 8 | 3 | 3 | 2 |
| *Falco tinnunculus (Linnaeus 1758)* | 5 | 1 | 3 | 1 |
| *Athene noctua (Scopoli 1769)* | 6 | 2 | 3 | 1 |
| *Bubo bubo (Linnaeus 1758)* | 7 | 2 | 3 | 2 |
| *Strix aluco (Linnaeus 1758)* | 6 | 1 | 3 | 2 |
| *Tyto alba (Scopoli 1769)* | 6 | 2 | 3 | 1 |
| **Common birds** | **5.59** | **1.71** | **2.70** | **1.17** |
| *Anas platyrhynchos (Linnaeus 1758)* | 5 | 1 | 3 | 1 |
| *Tachymarptis melba (Linnaeus 1758)* | 5 | 2 | 2 | 1 |
| *Caprimulgus europaeus (Linnaeus 1758)* | 5 | 2 | 2 | 1 |
| *Caprimulgus ruficollis (Temminck 1820)* | 5 | 2 | 2 | 1 |
| *Scolopax rusticola (Linnaeus 1758)* | 4 | 1 | 2 | 1 |
| *Columba livia (Gmelin 1789)* | 4 | 1 | 2 | 1 |
| *Columba oenas (Linnaeus 1758)* | 6 | 2 | 3 | 1 |
| *Columba palumbus (Linnaeus 1758)* | 5 | 1 | 3 | 1 |
| *Streptopelia turtur (Linnaeus 1758)* | 7 | 3 | 3 | 1 |
| *Alcedo atthis (Linnaeus 1758)* | 6 | 2 | 2 | 2 |
| *Clamator glandarius (Linnaeus 1758)* | 6 | 2 | 3 | 1 |
| *Alectoris rufa (Linnaeus 1758)* | 6 | 2 | 3 | 1 |
| *Coturnix coturnix (Linnaeus, 1758)* | 4 | 2 | 2 | 0 |
| *Fulica atra (Linnaeus 1758)* | 4 | 1 | 2 | 1 |
| *Gallinula chloropus (Linnaeus 1758)* | 4 | 1 | 2 | 1 |
| *Hippolais polyglotta (Vieillot 1817)* | 7 | 2 | 3 | 2 |
| *Calandrella brachydactyla (Leisler, 1814)* | 5 | 2 | 3 | 0 |
| *Galerida cristata (Linnaeus, 1758)* | 5 | 2 | 3 | 0 |
| *Galerida theklae (A. E. Brehm, 1857)* | 4 | 2 | 2 | 0 |
| *Lullula arborea (Linnaeus 1758)* | 7 | 2 | 3 | 2 |
| *Cettia cetti (Temminck 1820)* | 6 | 2 | 3 | 1 |
| *Cisticola juncidis (Rafinesque, 1810)* | 4 | 2 | 2 | 0 |
| *Corvus corax (Linnaeus 1758)* | 5 | 1 | 3 | 1 |
| *Corvus monedula (Linnaeus, 1758)* | 4 | 2 | 2 | 0 |
| *Garrulus glandarius (Linnaeus 1758)* | 6 | 1 | 3 | 2 |
| *Pica pica (Linnaeus 1758)* | 5 | 1 | 3 | 1 |
| *Pyrrhocorax pyrrhocorax (Linnaeus, 175* | 5 | 1 | 3 | 1 |
| *Emberiza hortulana (Linnaeus 1758)* | 7 | 2 | 3 | 2 |
| *Carduelis carduelis (Linnaeus 1758)* | 5 | 1 | 3 | 1 |
| *Chloris chloris (Linnaeus 1758)* | 6 | 1 | 3 | 2 |
| *Fringilla coelebs (Linnaeus 1758)* | 7 | 2 | 3 | 2 |
| *Linaria cannabina (Linnaeus 1758)* | 5 | 1 | 3 | 1 |
| **Species/Community** | **Relevance index** | **Degree of threat** | **Ecological interest** | **Expert criterion** |
| *Ptyonoprogne rupestris (Scopoli 1769)* | 6 | 2 | 3 | 1 |
| *Lanius collurio (Linnaeus, 1758)* | 5 | 2 | 3 | 0 |
| *Lanius meridionalis (Temminck 1820)* | 7 | 2 | 3 | 2 |
| *Lanius senator (Linnaeus 1758)* | 8 | 3 | 3 | 2 |
| *Locustella naevia (Boddaert, 1783)* | 4 | 1 | 3 | 0 |
| *Anthus campestris (Linnaeus 1758)* | 6 | 2 | 3 | 1 |
| *Anthus pratensis (Linnaeus, 1758)* | 5 | 2 | 3 | 0 |
| *Motacilla cinerea (Tunstall 1771)* | 6 | 2 | 3 | 1 |
| *Erithacus rubecula (Linnaeus 1758)* | 7 | 2 | 3 | 2 |
| *Luscinia megarhynchos (CL Brehm 1831)* | 7 | 2 | 3 | 2 |
| *Monticola saxatilis (Linnaeus, 1766)* | 4 | 2 | 2 | 0 |
| *Oenanthe hispanica (Linnaeus, 1758)* | 6 | 2 | 3 | 1 |
| *Cyanistes caeruleus (Linnaeus 1758)* | 5 | 2 | 2 | 1 |
| *Lophophanes cristatus (Linnaeus 1758)* | 6 | 2 | 3 | 1 |
| *Parus major (Linnaeus 1758)* | 7 | 2 | 3 | 2 |
| *Passer domesticus (Linnaeus 1758)* | 5 | 1 | 3 | 1 |
| *Passer montanus (Linnaeus 1758)* | 5 | 1 | 3 | 1 |
| *Petronia petronia (Linnaeus 1758)* | 6 | 2 | 3 | 1 |
| *Phylloscopus collybita (Vieillot 1817)* | 7 | 2 | 3 | 2 |
| *Regulus ignicapilla (Temminck 1820)* | 7 | 2 | 3 | 2 |
| *Regulus regulus (Linnaeus, 1758)* | 5 | 1 | 3 | 1 |
| *Sitta europaea (Linnaeus 1758)* | 7 | 2 | 3 | 2 |
| *Sturnus unicolor (Temminck 1820)* | 4 | 1 | 2 | 1 |
| *Sturnus vulgaris (Linnaeus 1758)* | 4 | 1 | 2 | 1 |
| *Sylvia atricapilla (Linnaeus 1758)* | 5 | 1 | 2 | 2 |
| *Sylvia cantillans (Pallas 1764)* | 5 | 1 | 2 | 2 |
| *Sylvia communis (Latham 1787)* | 6 | 2 | 3 | 1 |
| *Sylvia melanocephala (Gmelin 1789)* | 7 | 2 | 3 | 2 |
| *Sylvia undata (Boddaert 1783)* | 8 | 3 | 3 | 2 |
| *Turdus merula (Linnaeus 1758)* | 5 | 1 | 3 | 1 |
| *Turdus philomelos (CL Brehm 1831)* | 6 | 1 | 3 | 2 |
| *Turdus viscivorus (Linnaeus 1758)* | 6 | 1 | 3 | 2 |
| *Ardea cinerea (Linnaeus 1758)* | 6 | 3 | 2 | 1 |
| *Dendrocopos major (Linnaeus 1758)* | 7 | 2 | 3 | 2 |
| *Dendrocopos minor (Linnaeus 1758)* | 4 | 2 | 2 | 0 |
| *Dryocopus martius (Linnaeus 1758)* | 7 | 2 | 3 | 2 |
| *Jynx torquilla (Linnaeus 1758)* | 6 | 2 | 3 | 1 |
| *Picus viridis (Linnaeus 1758)* | 5 | 2 | 2 | 1 |
| **Chiroptera** | **6.67** | **2.14** | **3.00** | **1.52** |
| *Miniopterus schreibersii (Kuhl 1817)* | 8 | 3 | 3 | 2 |
| *Tadarida teniotis (Rafinesque 1814)* | 7 | 2 | 3 | 2 |
| *Rhinolophus euryale (Blasius 1853)* | 8 | 3 | 3 | 2 |
| *Rhinolophus ferrumequinum (Schreber 1774)* | 7 | 2 | 3 | 2 |
| *Rhinolophus hipposideros (Bechstein 1800)* | 6 | 2 | 3 | 1 |
| *Barbastella barbastellus (Schreber 1774)* | 8 | 3 | 3 | 2 |
| *Eptesicus (Eptesicus) serotinus (Schreber 1774)* | 7 | 2 | 3 | 2 |
| *Hypsugo savii (Bonaparte 1837)* | 7 | 2 | 3 | 2 |
| *Myotis blythii (Tomes 1857)* | 6 | 2 | 3 | 1 |
| *Myotis capaccinii (Bonaparte 1837)* | 7 | 3 | 3 | 1 |
| *Myotis emarginatus (E Geoffroy 1806)* | 6 | 2 | 3 | 1 |
| *Myotis escalerai (Cabrera 1904)* | 4 | 0 | 3 | 1 |
| *Myotis myotis (Borkhausen 1797)* | 7 | 3 | 3 | 1 |
| *Myotis nattereri (Kuhl 1817)* | 5 | 1 | 3 | 1 |
| *Nyctalus lasiopterus (Schreber 1780)* | 7 | 3 | 3 | 1 |
| *Nyctalus leisleri (Kuhl 1817)* | 7 | 2 | 3 | 2 |
| *Nyctalus noctula (Schreber 1774)* | 7 | 3 | 3 | 1 |
| *Pipistrellus (Pipistrellus) kuhlii (Kuhl 1817)* | 7 | 2 | 3 | 2 |
| **Species/Community** | **Relevance index** | **Degree of threat** | **Ecological interest** | **Expert criterion** |
| *Pipistrellus (Pipistrellus) pipistrellus (Schreber 1774)* | 7 | 2 | 3 | 2 |
| *Pipistrellus (Pipistrellus) pygmaeus (Leach 1825)* | 5 | 1 | 3 | 1 |
| *Plecotus austriacus (J Fischer 1829)* | 7 | 2 | 3 | 2 |
| **Small-mammals** | **5.57** | **1.43** | **2.71** | **1.43** |
| *Crocidura russula (Hermann 1780)* | 5 | 1 | 3 | 1 |
| *Arvicola sapidus (Miller 1908)* | 6 | 3 | 2 | 1 |
| *Myodes glareolus (Schreber 1780)* | 5 | 1 | 3 | 1 |
| *Eliomys quercinus (Linnaeus 1766)* | 7 | 3 | 3 | 1 |
| *Apodemus sylvaticus (Linnaeus 1758)* | 6 | 1 | 3 | 2 |
| *Mus (Mus) spretus (Lataste 1883)* | 5 | 0 | 3 | 2 |
| *Sciurus (Sciurus) vulgaris (Linnaeus 1758)* | 5 | 1 | 2 | 2 |
| **Common medium-size preys** | **5.50** | **1.50** | **2.50** | **1.50** |
| *Lepus (Eulagos) europaeus (Pallas 1778)* | 4 | 1 | 2 | 1 |
| *Oryctolagus cuniculus (Linnaeus 1758)* | 7 | 2 | 3 | 2 |
| **Ungulates** | **4.50** | **0.00** | **3.00** | **1.50** |
| *Capreolus capreolus (Linnaeus 1758)* | 4 | 0 | 3 | 1 |
| *Sus scrofa (Linnaeus 1758)* | 5 | 0 | 3 | 2 |
| **Carnivora** | **5.40** | **1.40** | **3.00** | **1.00** |
| *Vulpes vulpes (Linnaeus 1758)* | 4 | 0 | 3 | 1 |
| *Lutra lutra (Linnaeus 1758)* | 7 | 3 | 3 | 1 |
| *Martes foina (Erxleben 1777)* | 5 | 1 | 3 | 1 |
| *Mustela nivalis (Linnaeus 1758)* | 6 | 2 | 3 | 1 |
| *Genetta genetta (Linnaeus 1758)* | 5 | 1 | 3 | 1 |
| **Alien vertebrates** | **5.22** | **1.11** | **3.00** | **1.11** |
| *Cyprinus carpio (Linnaeus, 1758)* | 5 | 1 | 3 | 1 |
| *Gobio lozanoi (Doadrio & Madeira, 2004)* | 4 | 0 | 3 | 1 |
| *Phoxinus phoxinus (Linnaeus, 1758)* | 4 | 0 | 3 | 1 |
| *Gambusia holbrooki (Girard 1859)* | 7 | 3 | 3 | 1 |
| *Lepomis gibbosus (Linnaeus 1758)* | 6 | 2 | 3 | 1 |
| *Micropterus salmoides (Lacepède 1802)* | 6 | 2 | 3 | 1 |
| *Canis familiarus subsp. domesticus (Linnaeus 1758)* | 4 | 0 | 3 | 1 |
| *Felis catus (Linnaeus 1758)* | 5 | 1 | 3 | 1 |
| *Neovison vison (Schreber 1777)* | 6 | 1 | 3 | 2 |
