# Supplementary material for: A comprehensive but practical methodology for selecting biological indicators for long-term monitoring
Source: PLoS One. 2022 Mar 15;17(3):e0265246. doi: 10.1371/journal.pone.0265246 (PMC8923439; doi:10.1371/journal.pone.0265246)
Supplement: S2 Table — List of the 15 aggregated habitats (by similarity and type of plant formation) that collect in them the 96 habitats present in the Natural Park of Sant Llorenç del Munt i l’Obac. This list shows, the name of the aggregated habitat, the CORINE codes that are grouped in each aggregated habitat, and the description of the aggregated habitat. (DOCX) [file pone.0265246.s003.docx]

## S2 Table. List of aggregated habitats

List of the 15 aggregated habitats (by similarity and type of plant formation) that collect in them the 96 habitats present in the Natural Park of Sant Llorenç del Munt i l’Obac. This list shows, the name of the aggregated habitat, the CORINE codes that are grouped in each aggregated habitat and the description of the aggregated habitat.

| **Aggregated habitat** | **CORINE codes** | **Description** |
| --- | --- | --- |
| **Caves** | 65.4 | Caves, chasms and cavities. |
| **Cliff and crags** | 62.1111; 62.1115; 62.41; 62.12; 62.151; 62.152; 62.1C+; 62.41; 62.51 | Cliffs, crags and rock carvings of calcareous and siliceous conglomerates. |
| **Rocky lands** | 62.32+ | Pebbles, including extensive stony areas under the cliffs and mid-mountain. |
| **Dry meadows** | 34.36; 34.511; 34.5131; 34.634; 34.7133; 32.631+; 34.721; 34.722; 34.6322+; 35.31+; 35.32+ | *Brachipodium* grasslands and other formations of dry and calcareous soils. |
| **Crops** | 81.1; 82.11; 82.2; 82.31+; 82.32+; 82.33+; 83.11; 83.14; 83.182+; 83.182+; 83.211; 83.212; 83.221+; 83.3112; 83.3121; 83.3122; 83.3123; 83.321, 83.3251+; 83.322; 83.4+; 32.4A3; 34.6321+; 87.1 | Crops of herbs, fruit trees, vineyards, tree plantations and wastelands. |
| **Shrublands** | 31.8C2+; 31.8D; 31.8111; 31.8122; 31.82; 31.861; 31.863; 31.891; 32.1B+; 32.641+; 32.642+; 32.A; 32.4G; 32.B+; 32.1151+; 32.1152+; 32.322+; 32.335+; 32.346; 32.41; 32.42; 32.431; 32.433; 42.45, 32.4B+; 32.2121; 32.4C; 32.4D; 32.4E; 32.4F; 32.4H; 32.4811+ | Mediterranean and sub-Mediterranean groves, brushwood, garrigue, scrubland and maquis shrubland. |
| **Mediterranean pine forests** | 42.8412+; 42.8413+; 42.8414+; 42.8415+; 42.8416+; 42.8417+; 42.B4+ | Aleppo pine forest with a characteristic understory of scrub or maquis shrubland, ie without an oak forest understory. |
| **Humid pine forests** | 42.632; 42.67; 42.B3+; 42.5922+; 42.5B11+; 42.5E; 42.5F+ | Scots pine (*Pinus sylvestris*) and/or Pyrenean pine (*Pinus nigra* subsp. *salzmannii*) forests. |
| **Mixed forests** | 43.7131+; 43.7713; 43.H; 32.1121+; 32.1131+; 32.11611+; 45.3121+; 32.1134+; 45.345; 45.3123+; 45.3133+ | Forest formations with two tree layers of conifers and evergreens with similar dominances. Mediterranean holm oak forest is included to present a large number of pines in Sant Llorenç Natural Park. |
| **Mountain holm oak forests** | 32.1131+; 45.3132+; 45.321 | Mountainous oak forest characteristic of the upper part of the Sant Llorenç Natural Park. |
| **Deciduous forests** | 41.1751; 41.44; 41.2A+; 41.7131+; 41.7132+; 41.714; 41.7713; 41.9; 45.3122+; 45.3416+ | Oaks, mixed oak and holm oak forests and non-riparian deciduous forests. |
| **Riparian forests** | 24.224; 44.122; 44.124; 44.128+; 44.1412; 44.3432+; 44.6111+; 44.62; 44.637+ | Gray willow (*Salix atrocinerea*), white willow (*Salix alba*), silver poplar (*Populus* *alba*), elm (*Ulmus minor*) forests and other formations linked to watercourses. |
| **Freshwater** | 22.422; 22.441; 24.225; 24.43; 24.44; 54.12; 37.4; 54.12; 53.111; 53.112; 53.113; 53.62; 89.23 | Ponds, rivers, streams, springs, reed, reedbeds and others. |
| **Logged and/or burnt areas** | 90.1 | Recently disturbed environments (wildfires, forest felling, etc.). |
| **Urban** | 85.11; 85.12; 85.13; 85.14; 85.15; 86.1; 86.2; 86.3; 86.43; 86.21+; 86.22+; 86.24; 86.411; 86.412; 86.413; 86.42; 86.7+ | Loose or dense urbanized areas, parks and gardens, etc. |
