# Supplementary material for: A comprehensive but practical methodology for selecting biological indicators for long-term monitoring
Source: PLoS One. 2022 Mar 15;17(3):e0265246. doi: 10.1371/journal.pone.0265246 (PMC8923439; doi:10.1371/journal.pone.0265246)
Supplement: S3 Table — List of the valued habitats (aggregation by similarity and type of plant formation) of the Natural Park of Sant Llorenç del Munt i l’Obac following the criterion of achieving a value of the Relevance index greater than 4 points on a total of 8. The Relevance index of each selected grouping habitat corresponds to the average of the Relevance indexes of the different habitats that make up the group according to the selection criteria (relevance ≥ 4), being the sum of the four subsections to extract this index: degree of threat (scoring ranges from 0 to 2), the ecological interest (0 to 2), representativeness (0 to 2) and the expert or specialist criterion (0 to 2). Representativeness corresponds to the number of selected species that appear in the grouping habitat relativized on 2, being 380 the maximum of species assessed. (DOCX) [file pone.0265246.s004.docx]

## S3 Table. Selection values of habitats

List of the valued habitats (aggregation by similarity and type of plant formation) of the Natural Park of Sant Llorenç del Munt i l’Obac following the criterion of achieving a value of the Relevance index greater than 4 points on a total of 8. The Relevance index of each selected grouping habitat correspond to the average of the Relevance indexes of the different habitats that make up the group according to the selection criteria (relevance ≥ 4), being the sum of the four subsections to extract this index: degree of threat (scoring ranges from 0 to 2), the ecological interest (0 to 2), representativeness (0 to 2) and the expert or specialist criterion (0 to 2). Representativeness corresponds to the number of selected species that appear in the grouping habitat relativized on 2, being 380 the maximum of species assessed.

| **Grouping habitat** | **Relevance index** | **Degree of threat** | **Ecological interest** | **Representativeness** | **Expert or specialist criterion** |
| --- | --- | --- | --- | --- | --- |
| **Caves and potholes** | **4.1** | **2** | **1** | **0.08** | **1** |
| Cliff and crags | 3.4 | 1 | 1 | 0.38 | 1 |
| **Rocky areas** | **5.7** | **1** | **2** | **0.69** | **2** |
| Dry meadows | 3.7 | 2 | 1 | 0.70 | 0 |
| Crops | 1.7 | 0 | 1 | 0.67 | 0 |
| **Shrublands** | **4.8** | **1** | **2** | **0.79** | **1** |
| **Mediterranean pine forests** | **4.6** | **0** | **2** | **0.61** | **2** |
| **Wet pine forests** | **5.4** | **2** | **2** | **0.39** | **1** |
| **Mixed forests** | **4.5** | **0** | **2** | **0.54** | **2** |
| **Mountain holm oak forests** | **4.4** | **0** | **2** | **0.38** | **2** |
| **Deciduous forests** | **4.4** | **1** | **2** | **0.42** | **1** |
| **Riparian forests** | **5.6** | **2** | **2** | **0.59** | **1** |
| **Freshwater** | **6.3** | **2** | **2** | **0.35** | **2** |
| Logged and/or burnt areas | 3.6 | 0 | 2 | 0.63 | 1 |
| Urban | 1.5 | 0 | 1 | 0.49 | 0 |
