# Supplementary material for: A comprehensive but practical methodology for selecting biological indicators for long-term monitoring
Source: PLoS One. 2022 Mar 15;17(3):e0265246. doi: 10.1371/journal.pone.0265246 (PMC8923439; doi:10.1371/journal.pone.0265246)
Supplement: S4 Table — List of the valued ecosystem disturbances on Natural Park of Sant Llorenç del Munt i l’Obac following the criterion of achieving a value of the Relevance index greater than 2.5 points on a total of 5 (in bold). The list includes the ecosystem disturbances, the relevance index, and the three subsections to extract this index: the representativeness (scoring ranges from 0 to 2), the affected habitat (0 to 2), and the expert or specialist criterion (0 to 1). Representativeness corresponds to the number of selected species affected by the ecosystem disturbances relativized on 2, being 380 the maximum of species assessed. Habitat grouping is affected by the ecosystem disturbances relative to 2, with 11 being the maximum number of habitats assessed. (DOCX) [file pone.0265246.s005.docx]

## S4 Table. List and selection values of landscape and ecosystem disturbances

List of the valued landscape and ecosystem disturbances on Natural Park of Sant Llorenç del Munt i l’Obac following the criterion of achieving a value of the Relevance index greater than 2.5 points on a total of 5 (in bold). The list includes the change factor, the relevance index and the three subsections to extract this index: the representativeness (scoring ranges from 0 to 2), the effected habitat (0 to 2), and the expert or specialist criterion (0 to 1). Representativeness corresponds to the number of selected species affected by the change factor relativized on 2, being 380 the maximum of species assessed. Habitat grouping effected by the change factor relative to 2, with 11 being the maximum number of habitats assessed.

| **Change factor** | **Relevance index** | **Representativeness^1^** | **Habitat**^2^ | **Expert criterion** |
| --- | --- | --- | --- | --- |
| **Climatic**  **change** | **6.0** | **2.00** | **2.00** | **2** |
| **Wildfires** | **4.6** | **1.24** | **1.35** | **2** |
| **Alien species** | **3.2** | **0.41** | **1.84** | **1** |
| Forest pests | 1.5 | 0.11 | 1.35 | 0 |
| Animal diseases | 1.0 | 0.05 | 0.95 | 0 |
| **Afforestation** | **3.7** | **1.32** | **1.36** | **1** |
| Fragmentation | 2.0 | 0.96 | 1.05 | 0 |
| Erosion | 2.1 | 0.64 | 0.42 | 1 |
| **Freshwater alterations** | **3.0** | **0.49** | **0.48** | **2** |
| **Silvicultural exploitation** | **4.0** | **0.86** | **1.16** | **2** |
| Non-forestall products exploitation | 1.0 | 0.04 | 0.95 | 0 |
| **Hunting exploitation** | **3.7** | **0.07** | **1.58** | **2** |
| Freshwater fishing exploitation | 1.6 | 0.11 | 0.48 | 1 |
| **Human frequentation** | **3.1** | **0.92** | **1.22** | **1** |
| **Human infrastructures** | **4.4** | **0.54** | **1.84** | **2** |
