# Supplementary material for: A comprehensive but practical methodology for selecting biological indicators for long-term monitoring
Source: PLoS One. 2022 Mar 15;17(3):e0265246. doi: 10.1371/journal.pone.0265246 (PMC8923439; doi:10.1371/journal.pone.0265246)
Supplement: S1 Fig — The scheme used to select ecological processes in of the Natural Park of Sant Llorenç del Munt i l’Obac by a diagram of interactions between primary producers, food sources, primary consumers, secondary consumers, predators, decomposers, and abiotic and anthropogenic external ecosystem disturbances, such as climatology, perturbations, pollution, forest management, etc. (DOCX) [file pone.0265246.s006.docx]

## Figure S1. Selection of ecological processes

Scheme used to select ecological processes in of the Natural Park of Sant Llorenç del Munt i l’Obac by a diagram of interactions between primary producers, food sources, primary consumers, secondary consumers, predators, decomposers and abiotic and anthropogenic external factors, such as climatology, perturbations, pollution, forest management, etc.
