# Supplementary material for: A comprehensive but practical methodology for selecting biological indicators for long-term monitoring
Source: PLoS One. 2022 Mar 15;17(3):e0265246. doi: 10.1371/journal.pone.0265246 (PMC8923439; doi:10.1371/journal.pone.0265246)
Supplement: Attachment — Submitted filename: 211229_Review_Comments.docx [file pone.0265246.s007.docx]

**Journal Requirements:**

1. Please ensure that your manuscript meets PLOS ONE's style requirements, including those for file naming. The PLOS ONE style templates can be found at https://journals.plos.org/plosone/s/file?id=wjVg/PLOSOne_formatting_sample_main_body.pdf and https://journals.plos.org/plosone/s/file?id=ba62/PLOSOne_formatting_sample_title_authors_affiliations.pdf

We have followed PLOS ONE's style requirements

2. We note that Figure 3 in your submission contain map images which may be copyrighted. All PLOS content is published under the Creative Commons Attribution License (CC BY 4.0), which means that the manuscript, images, and Supporting Information files will be freely available online, and any third party is permitted to access, download, copy, distribute, and use these materials in any way, even commercially, with proper attribution. We require you to either (1) present written permission from the copyright holder to publish these figures specifically under the CC BY 4.0 license, or (2) remove the figures from your submission.

The parts of the figure that are sensitive to copyright have been replaced by the outlines drawn by the authors of the manuscript themselves.

**Reviewer #1:**

Thanks a lot for your valuable info that you provided in your targeted study and really sounds great for all the stockholders in the fields of biological and environmental science. Only you need to rewrite the abstract to be more clear and insert your aims of study also.

Many thanks for the review. We have tried to clarify our abstract.

**Reviewer #2:**

In their manuscript „a comprehensive but practical methodology for selecting biological indicators for long-term monitoring” the authors present how they selected a set of indicators to monitor changes in biodiversity at different levels in a Mediterranean ecosystem. The selection of suitable indicators is an important aspect to set up an effective monitoring system. The comprehensive approach is of interest to researchers facing the same challenge. This makes the paper a timely and important contribution. The paper is very readable. The tables and figures are informative and I found the supporting information good and well organized.

Many thanks for your point of view about our contribution to the selection of suitable indicators.

In the legend of figure 3 (L290) BMX appears twice (BMX: mixed forests AND BMX deciduous forests).

Thanks. We changed the abbreviation error.

The method presented in the manuscript is described by the authors as simple and practical. The steps in themselves are logical and easy to understand, however the exact execution is less clear. The authors build on a large amount of literature and prior research and their work flow includes the consultation of experts. Line 377 discusses the risk of undervaluing some components; line 392 the use of a qualitative method strictly based on preselected indicators. While the authors discussion of these points is relevant and comprehensible, they do contribute to the impression that the method is not as easy to implement as the authors initially imply.

Thanks for the comments. To improve the comprehensibility of our process we have improved the explanation from the 125 to 133 lines (Previously lines 119 to 146) and we have modified figure 1. Furthermore, we have rewritten and reordered the methods, especially State 3, State 4 and the new redaction of Results (sections 3.2 and 3.3).

The pre-requirements and the time aspect could be discussed further, especially as the aim of the method is to make monitoring more manageable.

Our method presents certain key steps that can compromise the good selection of indicators. Especially by the own intricacies of each taxon. For this reason, our method presents three evident prerequisites:

- Make an exhaustive collection of information and especially the most difficult, unpublished reports.
- Having knowledgeable naturalists in the area.
- To be able to consult specialists.

Then, understanding your concern, we have made some adjustments into State 2 of M&M;

New lines from 158 to 161: “An effective collection of prior information on the area to be applied, especially not published reports, is essential. If relevant prior information is not available, you need to back to State 1 to search for or generate relevant information”.

We also incorporate into the discussion, some new arguments about prerequisites (new lines from 394 to 399):

“Some possible biases could arise from the exclusive use of the weight values in the Relevance Index as some potential candidates (e.g. plants and invertebrate species) would be undervalued so expert criteria could be needed and this knowledge will reinforce the selection of candidates prioritized. It is a key of the scheme and process of selection to have a good information base beforehand (literature, reports and databases). The higher and better the quality of the information, the more rigorous the selection of components will be.”

This complements other phrases that emphasize the expert importance in the introduction, e.g. new line from 86 to 90: “[...] the knowledge of experts or specialists in local taxa is essential since one aspect of biodiversity (species, habitats, ecological processes, and biotic, abiotic and anthropic problems) may affect a focal region differently and so require its indicator [6]. It is also important to assess which indicators are valid and informative for a region and which are redundant, overvalued or unnecessary”.

Moreover, in methods, e.g. new lines from 198 to 200: "[...] the bibliography and expert criteria are essential (Table 2), and will help highlight species or habitats that are not present or not evaluated as threatened [...]".

Some of the aspects of the methodology, specifically the step from biodiversity components to indicators, were not entirely clear to me. The methodology does not sufficiently distinguish between parameters (species, habitats, processes, factors) and the assessment methods (abundances, phenology etc.). For instance, line 233 gives butterflies as an example, but does not specify an actual parameter that is measured.

To facilitate the discrimination between parameters and method, we reformulate the new lines 228 to 244, of the State 4: Monitoring Catalogue section of the document:

“Once the different relevant components have been assessed and selected, a Monitoring Catalogue (Figure 1) can be established to act as a strategic guide, facilitate understanding, and identify which variables need to be sampled and measured to generate the biological indicators in an ecological sense and to avoid redundancies. To attain the list of relevant components, (1) we grouped components by taxonomic, physiognomic, and functional similarities (e.g. common birds, freshwater invertebrates, decomposers). Then, (2) classified the grouped relevant components in four ecological levels: species, habitat, ecological process, and ecosystem disturbances (see the case study below as a practical example). (3) For each group of relevant components we selected the adequate variables to be sampled, e.g., species presence and abundance, reproductive and survival taxes, habitat preferences, soil structure, and composition. Lastly, (4) incorporated into each level all the monitoring components whilst bearing in mind that each can be part of one or more levels depending on their role and interactions within the ecosystem (see State 3). For example, species abundance and richness of butterflies provide information about the species level and their community (indicators at species level), about the variation on ecosystem functional capabilities, e.g., as primary consumers (indicators at ecological process level), or climate change and afforestation effects (indicators at ecosystem disturbance level)”

On page 8 and 9, the authors set a threshold of >= 4 for their index. I was unsure how this threshold was set and of its implications for the selection of species.

We understand the question raised. Here we tried to establish a criterion that was rigorous and, at the same time, did not rule out underrepresented components. For this purpose, the criterion was half of the Relevance index. Thus, this criterion was chosen to greatly reduce the number of initial components but, at the same time, to ensure that those assessed by the experts were not lost.

We have incorporated the following explanation in new lines 198 to 193 of the State 3: The establishment of the relevant components of monitoring biodiversity selection for clarification:

“Because the threshold value needs to be sufficiently robust but at the same time to include potential under-detected components, we established as a criterion to maintain the components with a Relevance index equal to or above half of the maximum global potential value that included valuable ecosystem components, as we have verified in our previous tests (see Table 1).”

In Table 2, “ecological process” stood out to me as a criterion: the description could apply to all species.

Here, the selection criterion corresponding to the description was applied to all species. Unfortunately, this information is not known in-depth for all species, especially invertebrates.

The methodology that allowed to group the 434 relevant components into 32 specific monitoring components requires more details.

We have redefined the section State 4: Monitoring Catalogue section (new lines from 228 to 244) of the document for clarification, and wrote all grouped components:

“Once the different relevant components have been assessed and selected, a Monitoring Catalogue (Figure 1) can be established to act as a strategic guide, facilitate understanding, and identify which variables need to be sampled and measured to generate the biological indicators in an ecological sense and to avoid redundancies. To attain the list of relevant components, (1) we grouped components by taxonomic, physiognomic, and functional similarities (e.g. common birds, freshwater invertebrates, decomposers). Then, (2) classified the grouped relevant components in four ecological levels: species, habitat, ecological process, and ecosystem disturbances (see the case study below as a practical example). (3) For each group of relevant components we selected the adequate variables to be sampled, e.g., species presence and abundance, reproductive and survival taxes, habitat preferences, soil structure, and composition. Lastly, (4) incorporated into each level all the monitoring components whilst bearing in mind that each can be part of one or more levels depending on their role and interactions within the ecosystem (see State 3). For example, species abundance and richness of butterflies provide information about the species level and their community respectively, so in the latter case indicate the variation on ecosystem functional capabilities (e.g. as primary consumers), climate change or afforestation effects. ”

Ant the 3.3. Monitoring catalogue and Priority indicators selection (new lines from 355 to 373):

“To constitute the Monitoring catalogue, we grouped the 434 Relevant components independently by the level of species, habitats, ecological processes, and ecosystem disturbances by similarities. In the case of 408 species were grouped by taxonomic, physiognomic, and functional similarities into 17 groups of species (threatened plant species, xeric gastropods, granivorous ants, decomposers, orthoptera, butterflies, freshwater macroinvertebrates, freshwater fishes, amphibians, reptiles, common birds, raptors, bats, small mammals, common medium-size preys, ungulates and carnivores). A total of 96 habitats were grouped in 11 habitat relevant categories (Table 4). Then, the habitat category resulted in two monitoring parameters (habitat structure and composition), which will be carried out in the 11 selected relevant habitats. Moreover, it is in these habitats where the specific transversal monitoring of species, processes and disturbances will be developed. Ecological processes were grouped by ecological functions in 6, decomposition, primary production, consumers, predators, zoocory, and pollination. Ecosystem disturbances were maintained in 8 (Table 4), climatic change, wildfires, afforestation, freshwater alterations, alien species, exploitation of natural resources, forests pests, human frequentation, human infrastructures). So resulted in a total of 32 specific Monitoring Components. Finally, to obtain the Priority Indicators we used the quantitative Priority Index, and 27 of these Monitoring Components were selected to monitor the biodiversity in the PNSLL (Table 5).”

Relevant components selection: I’m not sure about the grouping of species: while I understand that the % of alien species is of interest, they could also fit into the taxonomic groups.

Although the reviewer is right (alien species could be grouped into the respective taxonomic groups), in previous discussions with managers and experts, we decided to give a category for relevant alien species, as an independent indicator. However, they are noted in surveys when autochthonous species (e.g. vegetation) are noted, i.e., they do not need a special survey. However, Then, in this case, as in others, the same species indicates on more than one level: as an indicator of species status, as well as an indicator of the landscape and ecosystem disturbances (as biological invasion).

I think it would be more easily understandable to provide the numbers separately (classification into organism groups and a separate % for status). It might also be more easy to give species numbers rather than % - please check the numbers, as the % in the text do not always seem to match up with the number of species given in Annex 2.

Thanks, we reviewed the numbers.

Reviewer suggestion was incorporated on 3.2. Relevant components selection section (new lines from 314 to 323):

“The relevant components were obtained through a Relevance Index which was calculated for all 3,226 assessed species (Table 4) of which 406 (12.6%) were selected as Relevant Components (S1 Table): plants contributed with 69 species (17% - of which 10 species were threatened species, 13 pteridophytes, 41 vascular plants, and five alien species), 191 species (47%) were invertebrates (of which 26 species were Odonata, 12 Gastropoda, 43 decomposers, 22 Formicidae, 30 Orthoptera; 36 Lepidoptera, ten other invertebrates of special interest, seven plague species and five were alien species), while 146 species (36%) were vertebrates (of which tree species were freshwater fish, seven amphibians, seven reptiles, 13 raptors, 70 common birds, 21 bats, seven small-mammals, two common medium-sized preys, two ungulates, five carnivores and nine were alien species)”

Thanks, we reviewed the numbers.
