# Supplementary material for: A comprehensive but practical methodology for selecting biological indicators for long-term monitoring
Source: PLoS One. 2022 Mar 15;17(3):e0265246. doi: 10.1371/journal.pone.0265246 (PMC8923439; doi:10.1371/journal.pone.0265246)
Supplement: Attachment — Submitted filename: 220221_Editor_Comments.docx [file pone.0265246.s008.docx]

**Academic Editor:**

I acknowledge the huge effort performed to summarise and synthetize all the information recorded, in such an easy way to be processed by the readers. However, there are still some points to be addressed prior to acceptance:

Line 174: include references for “monitoring programs”* and “expert criterion”

*Torre, I., López-Baucells, A., Stefanescu, C., Freixas, L., Flaquer, C., Bartrina, C., Coronado, A., López-Bosch, D., Mas, M., Míguez, S., Muñoz, J., Páramo, F., Puig-Montserrat, X., Tuneu-Corral, C., Ubach, A., Arrizabalaga, A., 2021. Concurrent Butterfly, Bat and Small Mammal Monitoring Programmes Using Citizen Science in Catalonia (NE Spain): A Historical Review and Future Directions. Diversity 13, 454. doi:10.3390/D13090454

Thanks for the reference, we have added the paper to the references. On the other hand, we have included “Duelli P, Obrist MK. Biodiversity indicators: the choice of values and measures. Agriculture, ecosystems & environment. 2003; 98(1):87-98. doi: 10.1016/S0167-8809(03)00072-0” for the expert criterion.

The scale values assigned to each criterion need to be further justified. Why “Threat” is in a scale 0-3, “Ecological Interest” in a scale 0-2, and “presence on monitoring programs” in scale 0-1? Why assigning more weight to Threat than to Ecological Interest? This represents a subjective starting point decided by authors.

Thanks for your comment. Degree of threat and Ecological Interest were to be given equal weight in the selection process. In the specific case of species, initially the "presence on monitoring programs" count as part of Ecological interest, as they correspond to common species (commonly detected species in monitoring plans). However, to avoid the misunderstandings and subjectivity that this implies, we have placed this category ("presence on monitoring programs") within Ecological interest. In this way, Ecological interest and Degree of threat have equal weight (from 0 to 3) in the final model (see Table 1, Table 2, and Table S1).

This is well explained in the new lines 76-90, 197-204 and 406-422 of the manuscript. However, we have also modified the following sentence in the new line 201-202 of the manuscript, to facilitate understanding:

“The ecological interest contributes to minimize the weight of these threatened and singular component, which do not necessarily reflect the spatial and temporal trends of biodiversity [23, 55] but, rather, respond to the local conservation status”

In supporting Table, I see some data that not match my knowledge on the species selected. Some small mammals having monitoring programs, scored “0”. Also, expert criterion is low for species with reduced populations (*M.glareolus, E.quercinus*), so it’s disappointing. This needs to be corrected or justified. I don’t know if such differences are affecting to other taxonomic groups. This will affect final scores, and need to be recalculated, if necessary.

In the first case, the criterion was to assign a “1” if the species was detected previously in a specific monitoring program. Due to *E.quercinus* was captured by the monitoring program in 2018 and *M.glareolus* has the potential to be captured by SEMICE monitoring program; have updated the assessment of the two species. However, we have not updated the rest of the species since in the monitoring that has been carried out since 2018 (the database used here was last updated in 2017) in the natural park there have not been any new records, see Puig-Gironès R. 2020 *Catàleg de Flora Vascular, Fauna Invertebrada i Fauna Vertebrada del Parc Natural de Sant Llorenç del Munt i l’Obac. Diputació de Barcelona, Barcelona.*

The document presented is not intended to be an immovable document, and it is proposed to be re-evaluated in a couple of years (2024 or 2025). Therefore, we have decided not to modify the evaluation of the expert criterion since this revaluation will be made for the totality of the species. Experts and specialists were consulted to evaluate the ecological and conservation interest of each species (see acknowledgements for the list of the consulted experts), and they examined each biodiversity component and judged their importance in the focal region to minimise the risk of benefiting certain charismatic taxa or a predetermined species as an indicator.

Also raised by reviewers…Why a threshold is set in the mean, >=4 ? A rank criterion, ordering the species from 0-8, will be better since between 5-8 there are significant differences of suitability that could help selecting in particular situations (ex. limited resources). If one wants to select the most relevant indicators, I will choose those having 8 scores.

Thanks for the comment. We understand the reasoning of the editor and reviewers. However, the aim of this paper, as detailed in the introduction and discussion, is to be a balanced method (see again, the new lines 76 – 90, 197-204 and 406-422).

Given the dissimilarities in the knowledge and subsequent assessment of the species, to put a very high threshold as 8 entail biased indicators to more visible, detectable and studied species. For example, selecting those species with a Relevance index of 8, eight of the ten species of threatened and endemic flora would not be chosen, such as: *Arenaria conimbricensis* subsp. *conimbricensis*, *Arenaria fontqueri* or *Erodium glandulosum*, nor the endemic gastropods like *Montserratina bofilliana*, *Xerocrassa montserratensis* or *Abida secale* subsp. *bofilli*.

On the other hand, if the threshold were ≥ 7, then 57 species would be selected, of which: 8 would be plants (14%), 5 invertebrates (8.8%) and 44 vertebrates (77.2%) so at first instance represents a high biased frequency compared to listed plants (32%), invertebrates (61%) and vertebrates (7%) in the Sant Llorenç del Munt Natural Park (see Puig-Gironès R. 2020 *Catàleg de Flora Vascular, Fauna Invertebrada i Fauna Vertebrada del Parc Natural de Sant Llorenç del Munt i l’Obac*. Diputació de Barcelona, Barcelona).

These enormous differences are also maintained in the threshold ≥ 6 and 5. Therefore, we set the threshold at ≥ 4, since this limit allowed us to have reasonable percentages between plants, invertebrate and vertebrate species to follow as indicator.

Expert criteria seem a rather subjective selection criterion. Indeed, experts use similar criteria for selecting indicators regarding threat and ecological interest of the species, so it could be a redundant variable. Furthermore, a “0” indicates that experts consider the species irrelevant, or there is no information available?

Our main objective was a collaborative design process to enhance the acceptance of diverse values and prioritizations embedded in sustainability assessments. This emphasis on the process would make assessments more transparent, transformative and enduring. Even so, in certain species, habitats or processes there may be some redundancy, but we believe that the expert criterion based on the local scale minimized this effect. Furthermore, given the lack of monitoring systems or the lack of catalogues of endangered species (in the case of Catalonia we do not have a Catalogue of Endangered Fauna), expert judgement fills this gap.

On the other hand, expert criterion is necessary to assess which indicators are valid and informative for a region. As detailed in Table 2: “the criterion of external expert or taxon specialist grants a value of 2 to the species, habitats, and/or ecosystem disturbances with high importance and relevance in the context of the study area, and 1 to species, habitats, and/or ecosystem disturbances with relative importance in the study area”.

Table 1. why “z”? It should correspond to “f”. Please, justify.

Thanks to finding the error, we have changed 'z' to 'f'.

Table 2. Correct “Specie” to “Species”

Thanks, we have fixed the error.
